# Supplementary figures and images for: CLIC1 down-regulates Nrf2/HO-1 signalling pathway promoting the apoptosis and pyroptosis in OGD/R-treated HT22 cells
Source: PLoS One. 2025 Sep 18;20(9):e0332698. doi: 10.1371/journal.pone.0332698 (PMC12445528; doi:10.1371/journal.pone.0332698)

Figure 2c

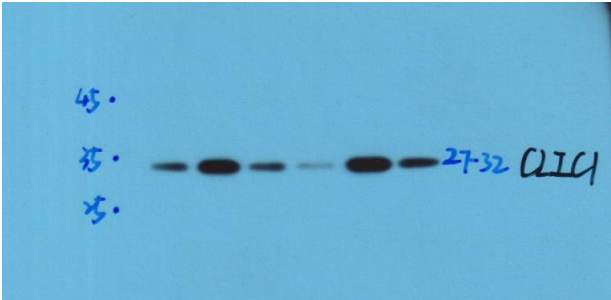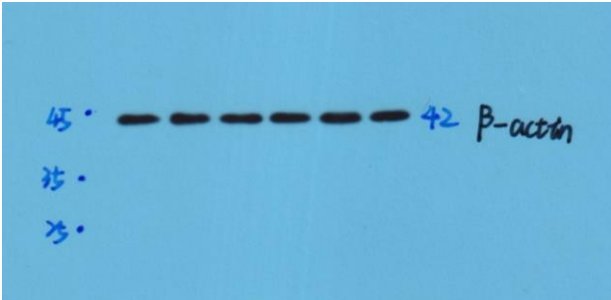

(1)

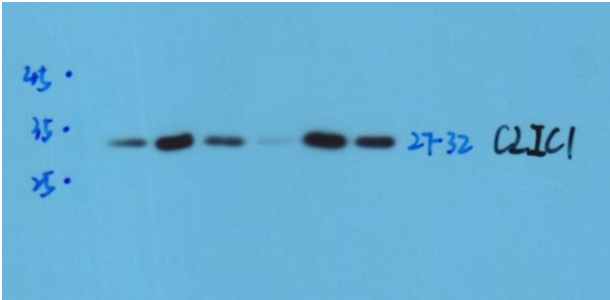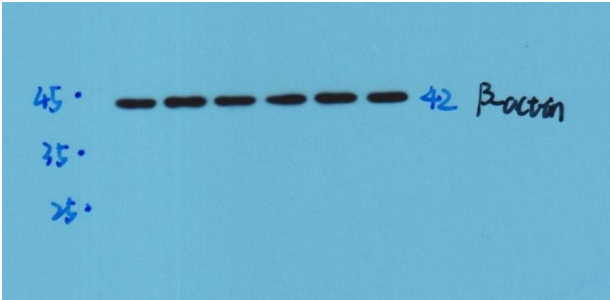

(2) ✓

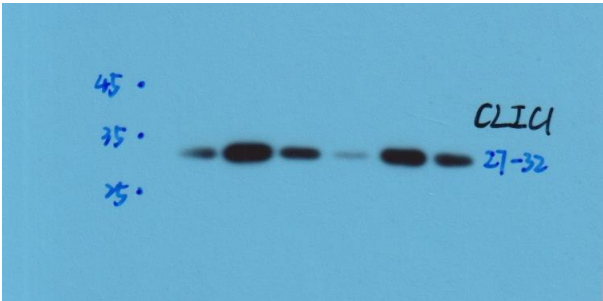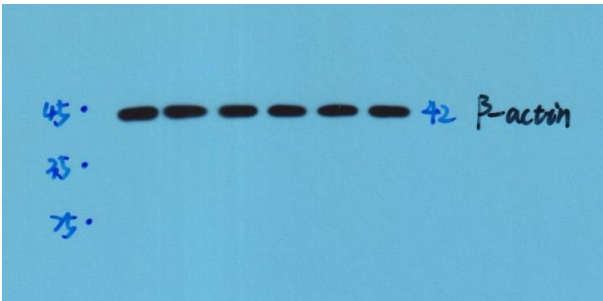

(3)

Figure 3h

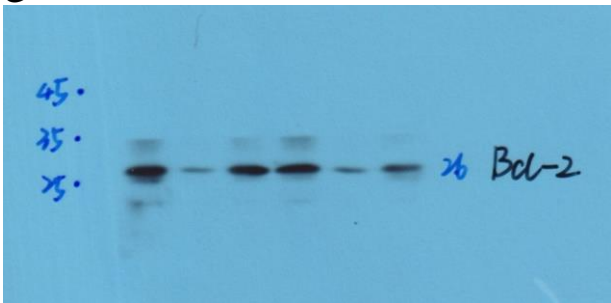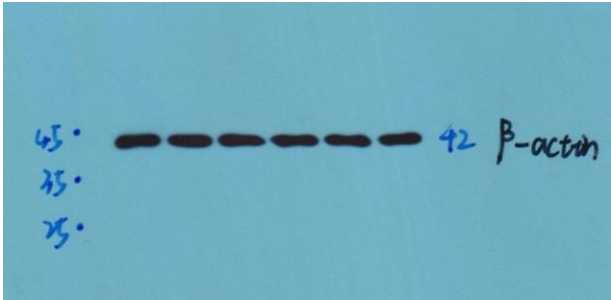

(1)

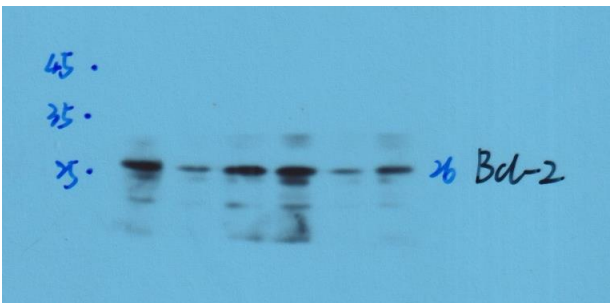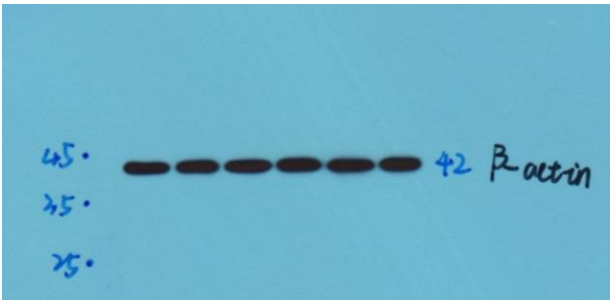

(2)

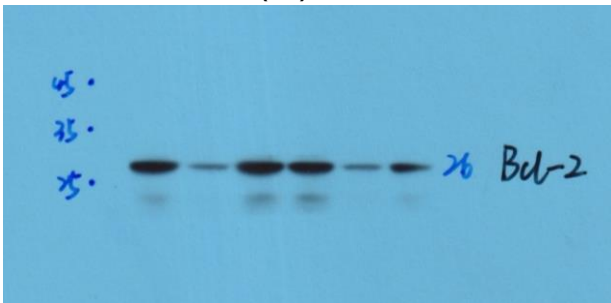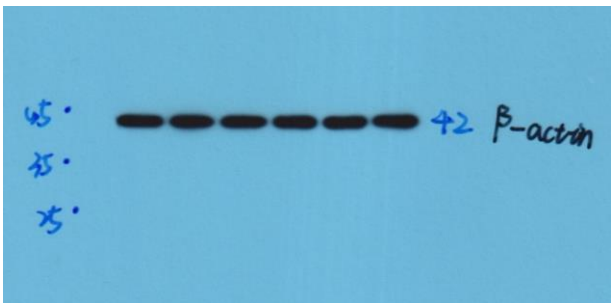

(3) ✓

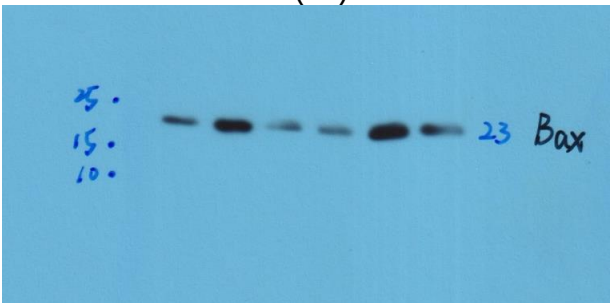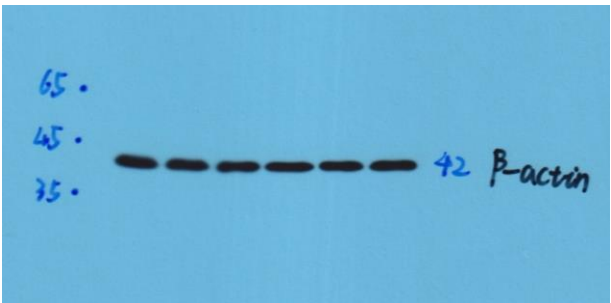

(1)

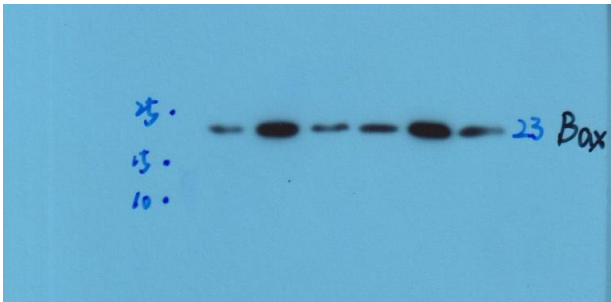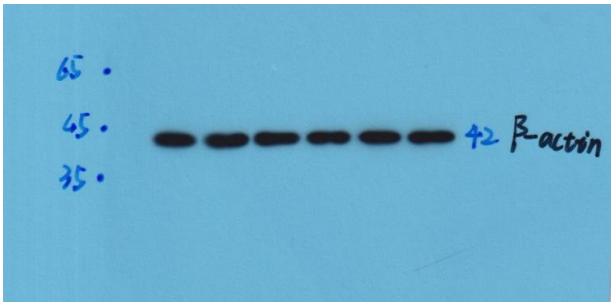

(2) ✓

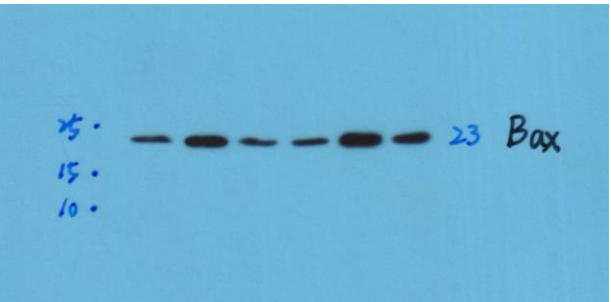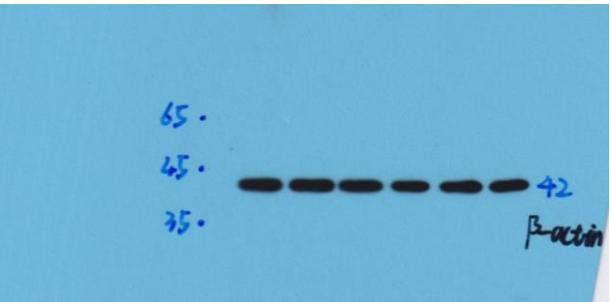

(3)

Figure 4c

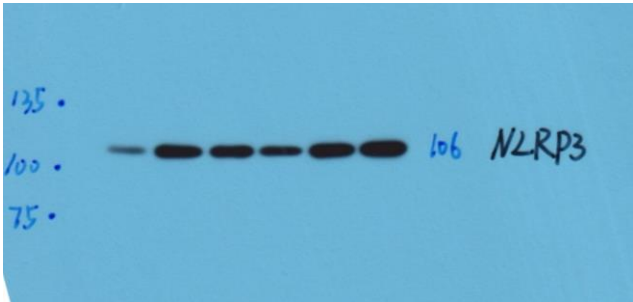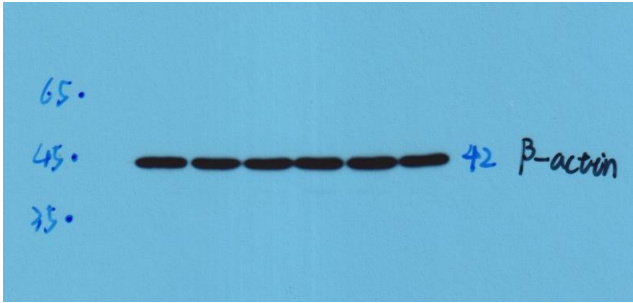

(1)

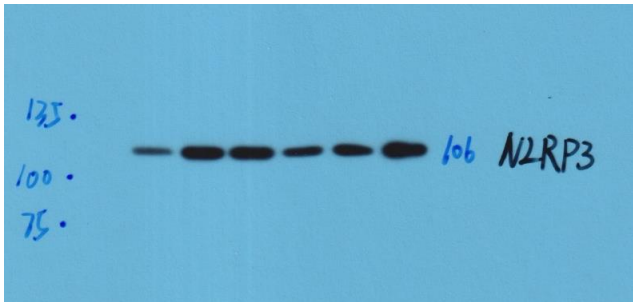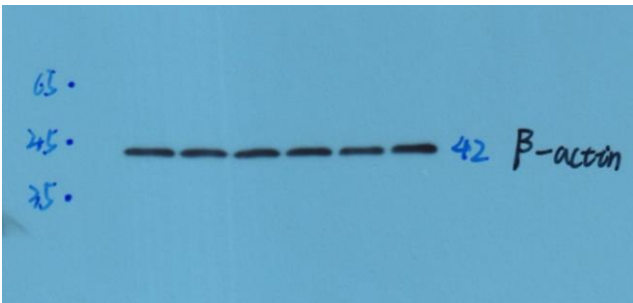

(2)

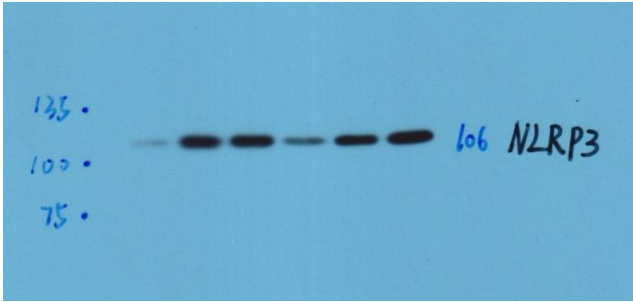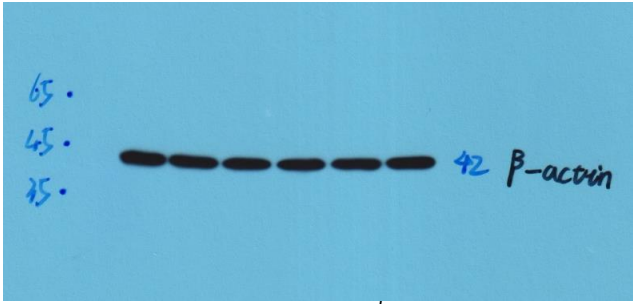

(3) ✓

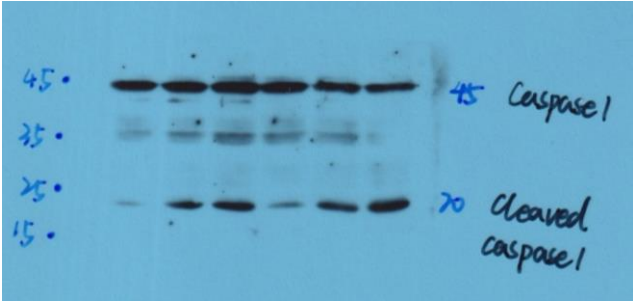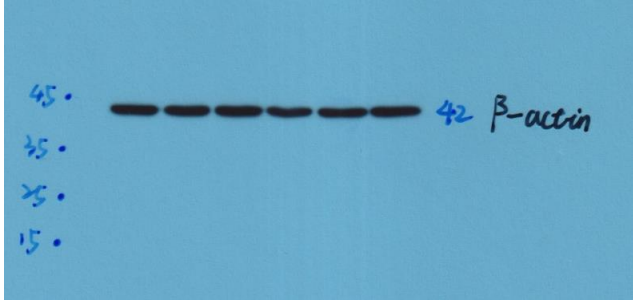

(1)

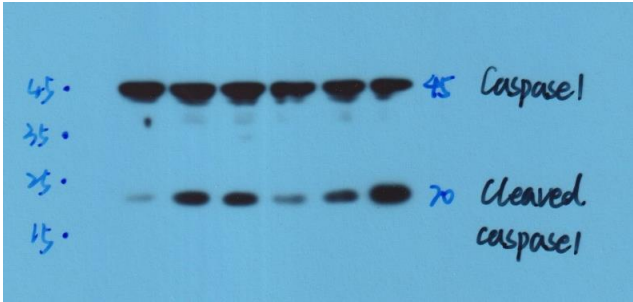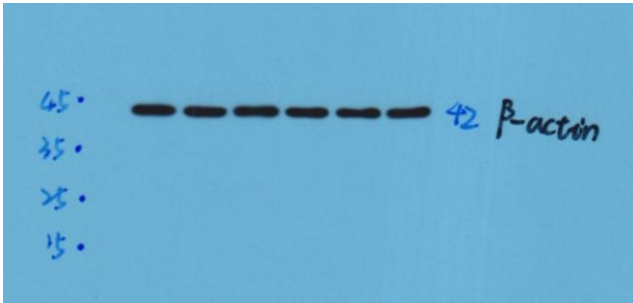

(2)

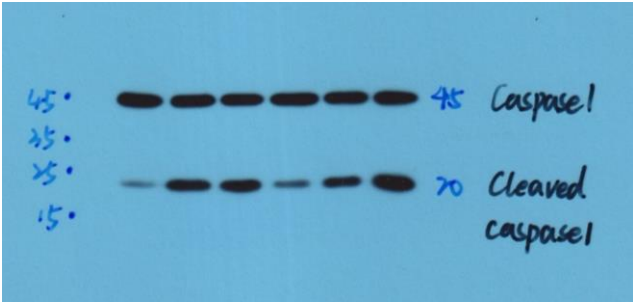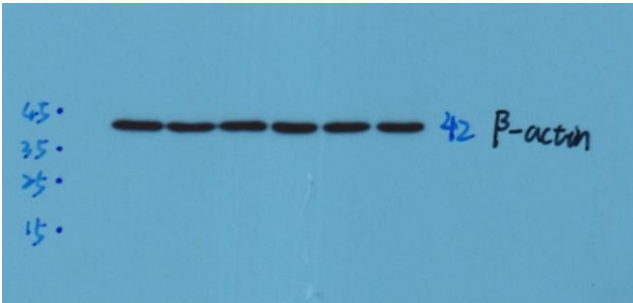

(3) ✓

Figure 4f

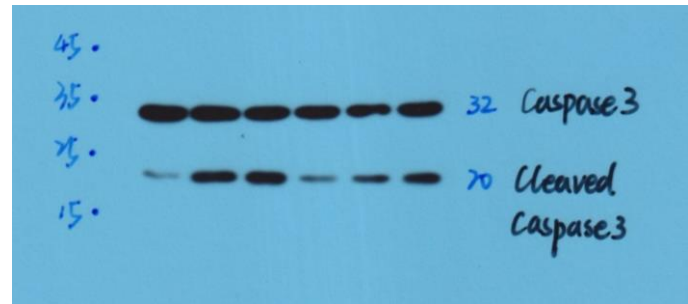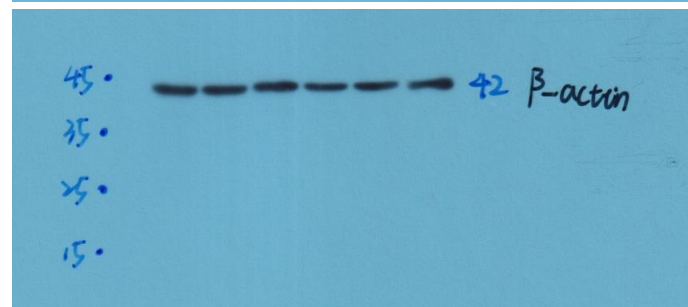

(1)

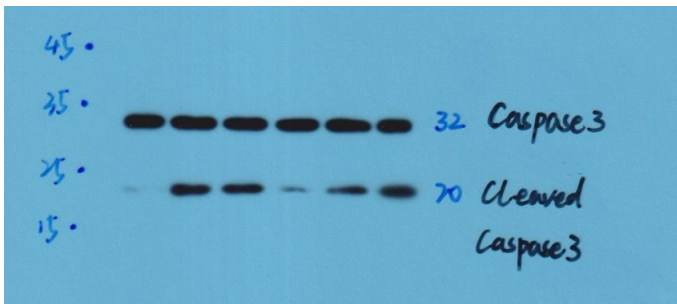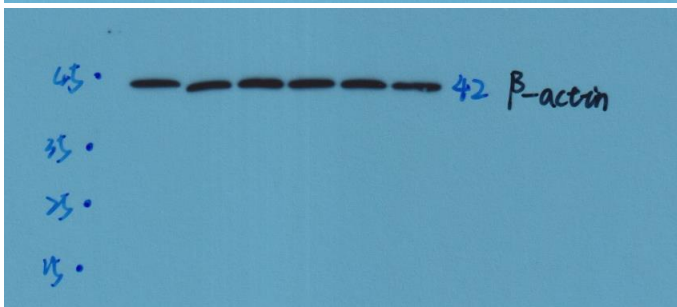

(2)

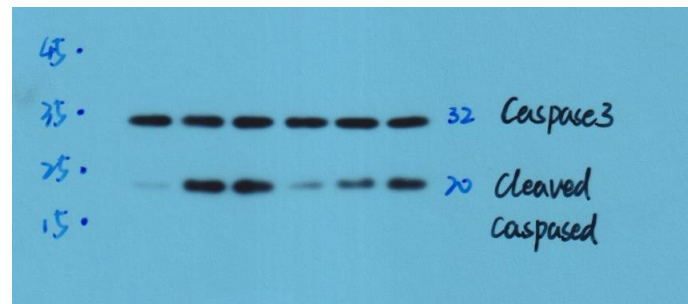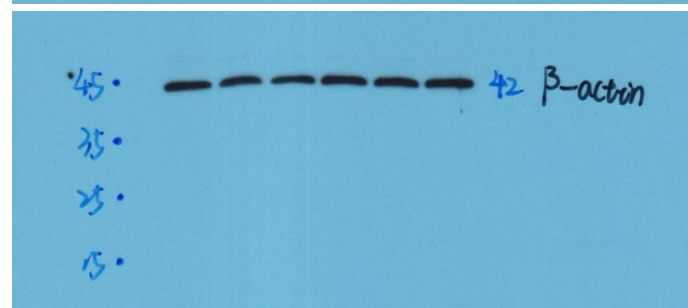

(3)  $\checkmark$

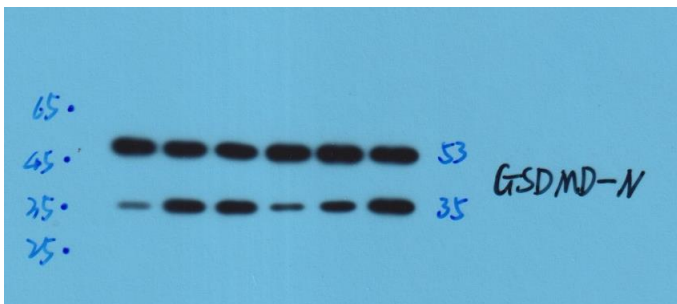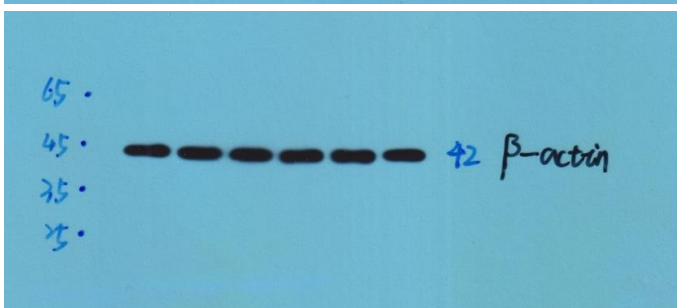

(1)

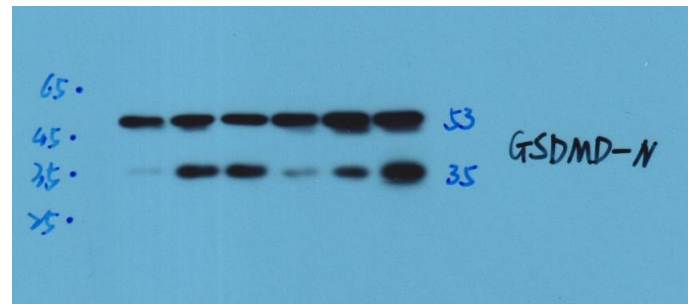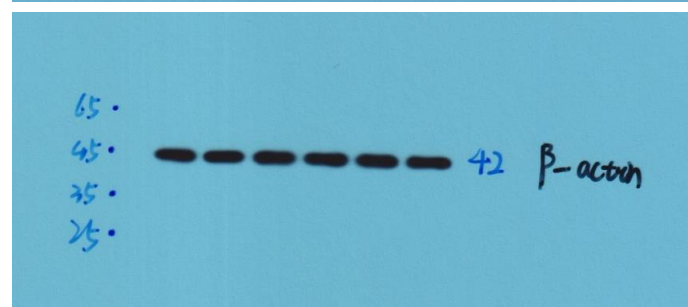

(2)  $\checkmark$

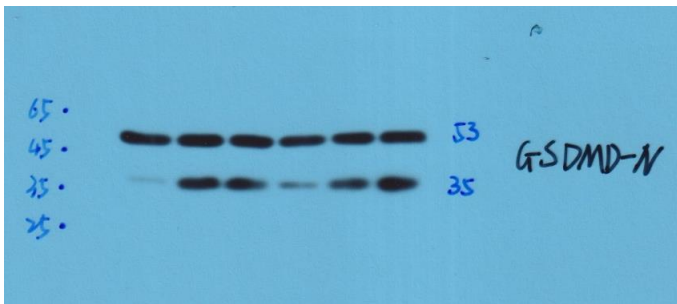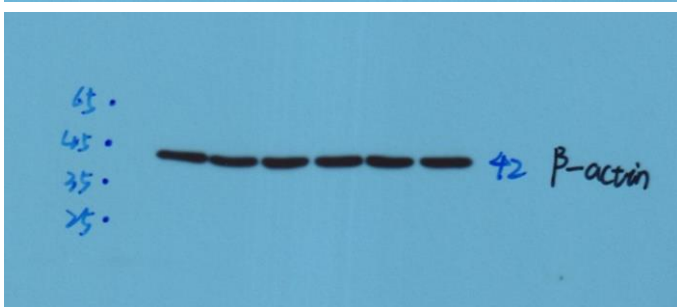

(3)

Figure 4i

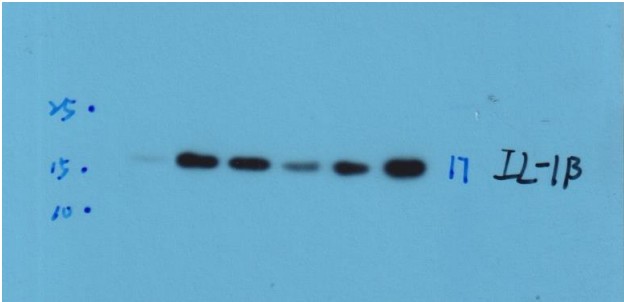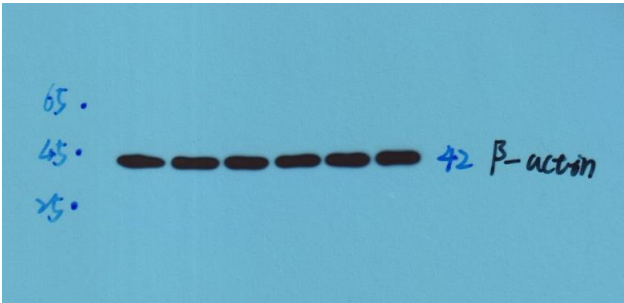

(1)

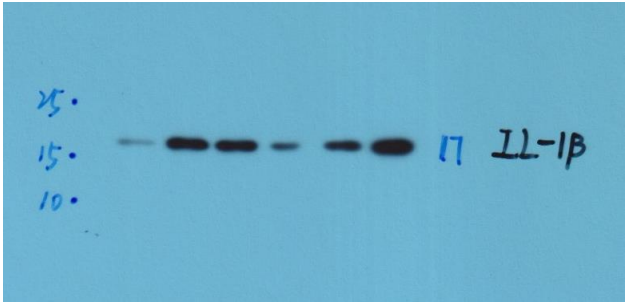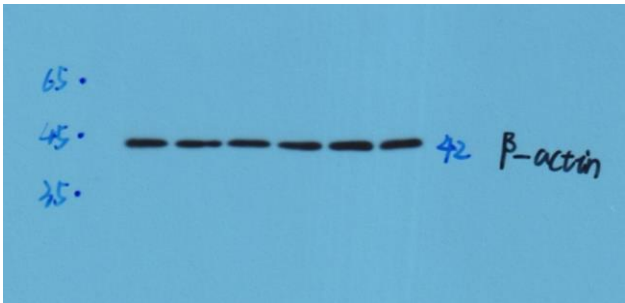

(2)

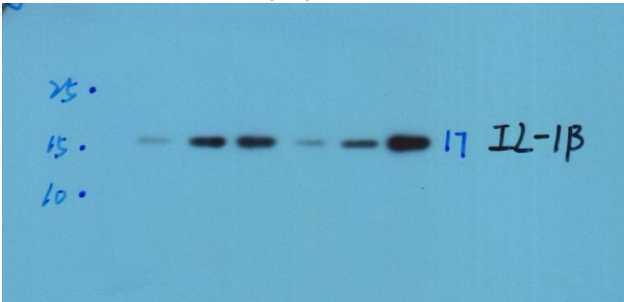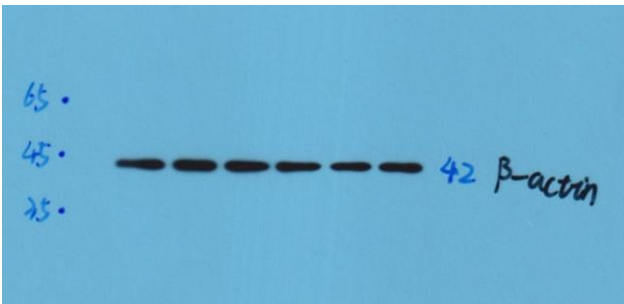

(3) √

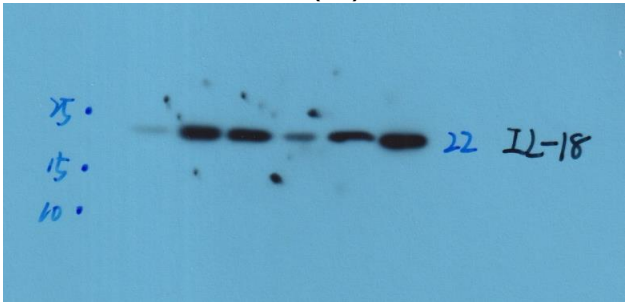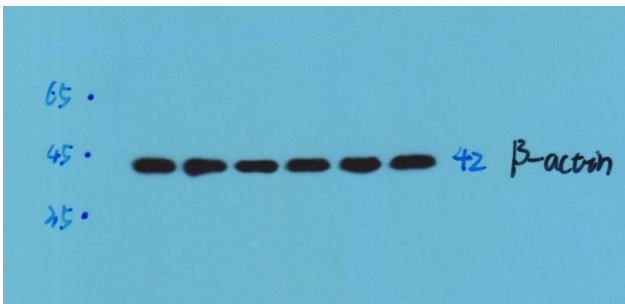

(1)

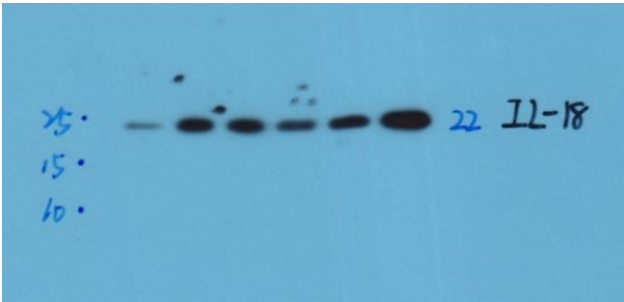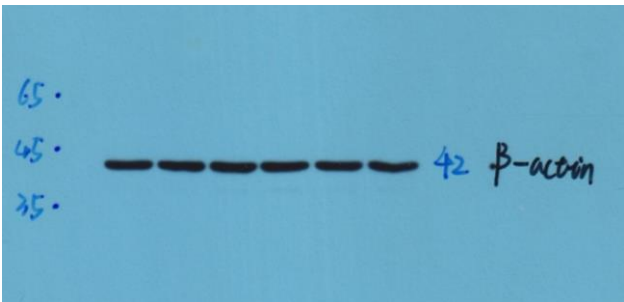

(2) √

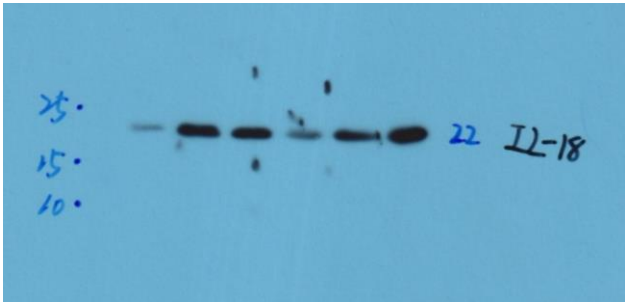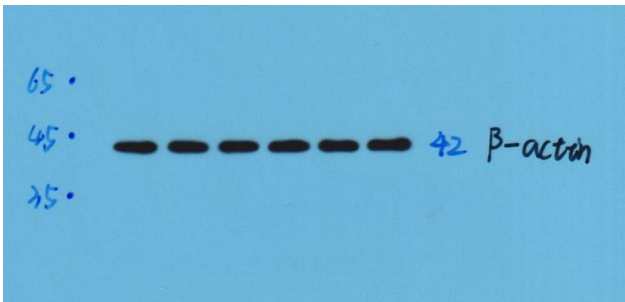

(3)

Figure 5c

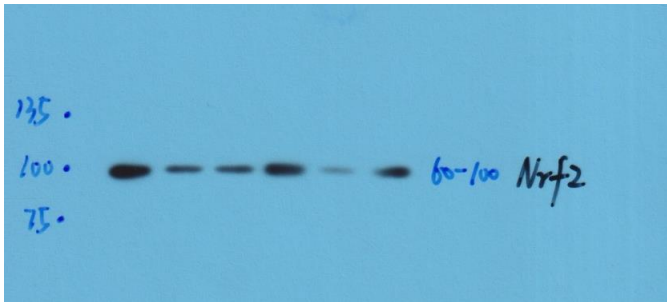

(1)

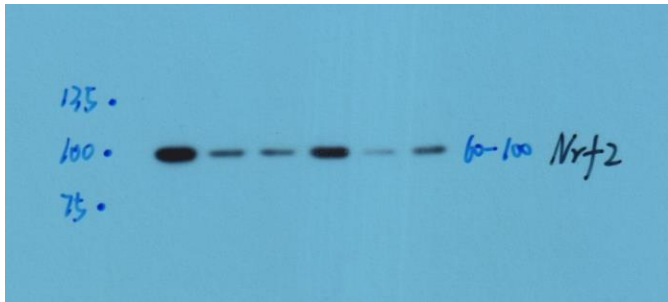

(2) √

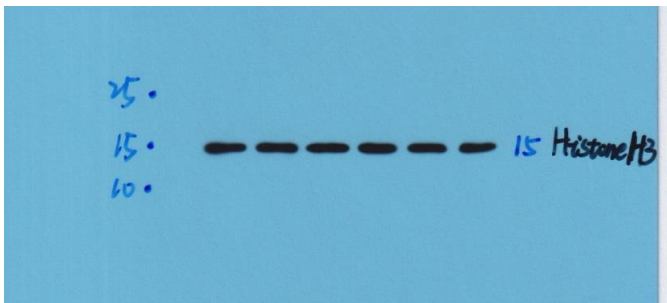

(3)

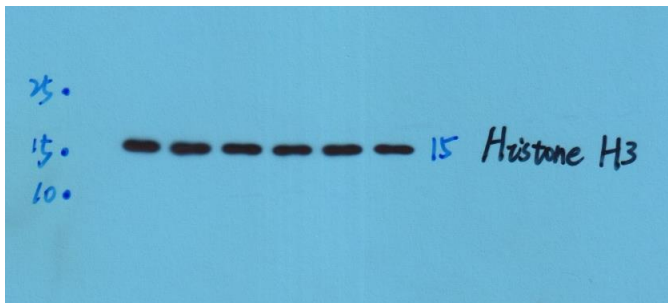

(1)

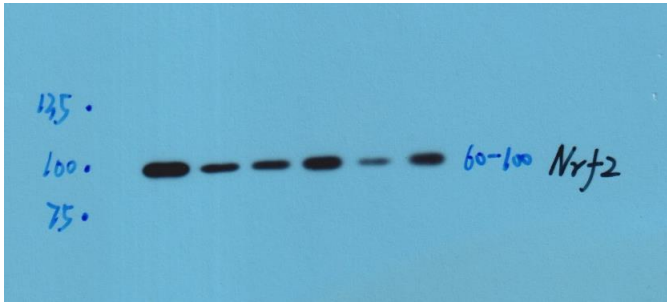

(2) √

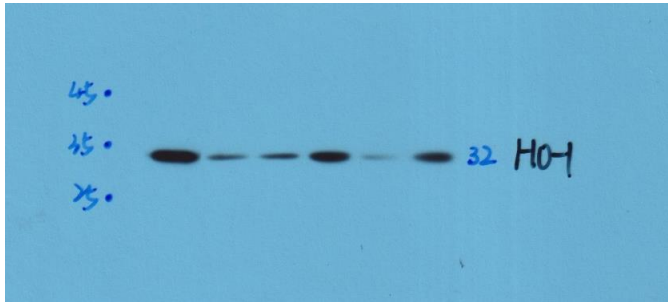

(3)

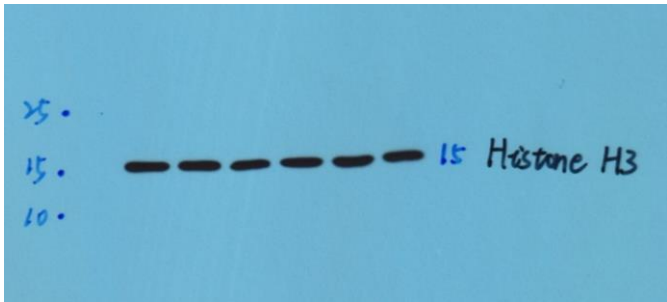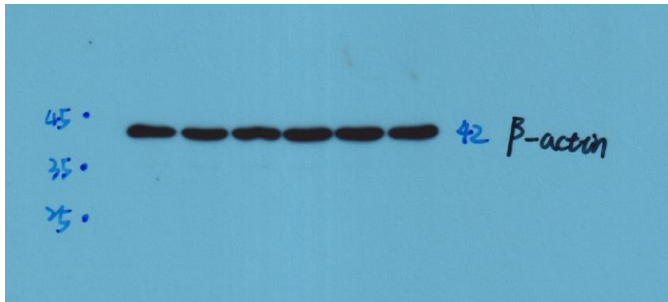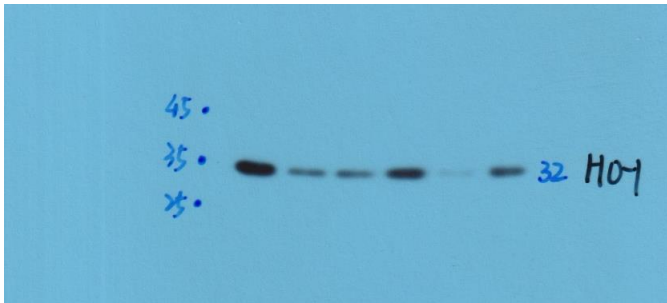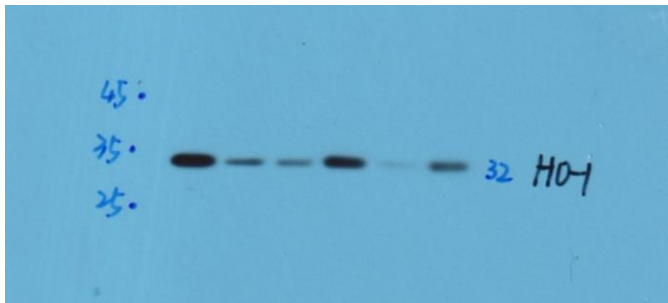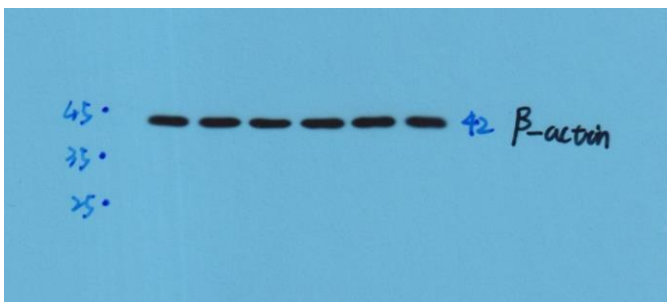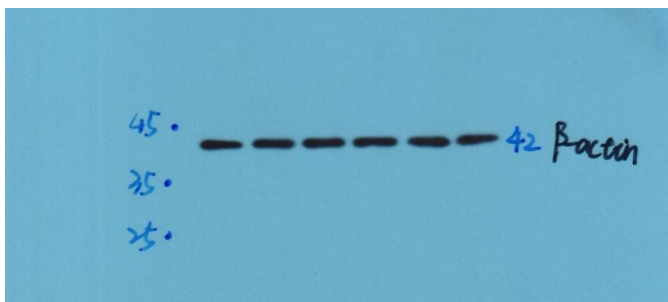

Figure 7h

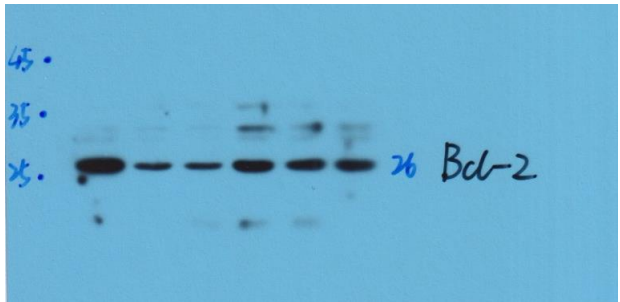

(1)

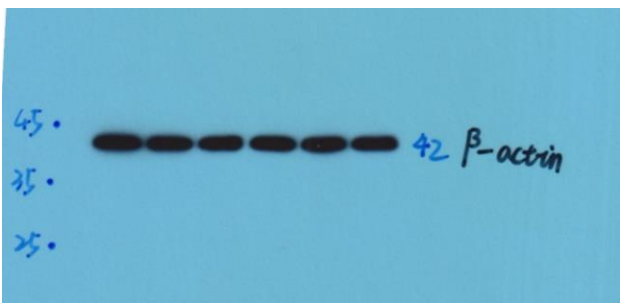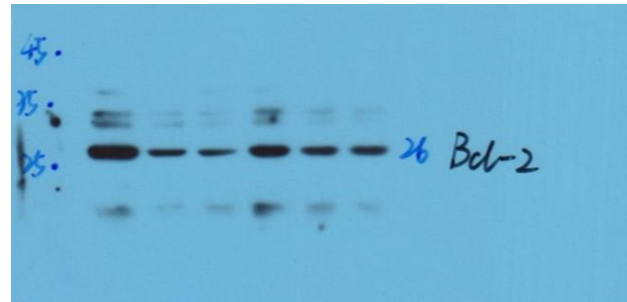

(2)

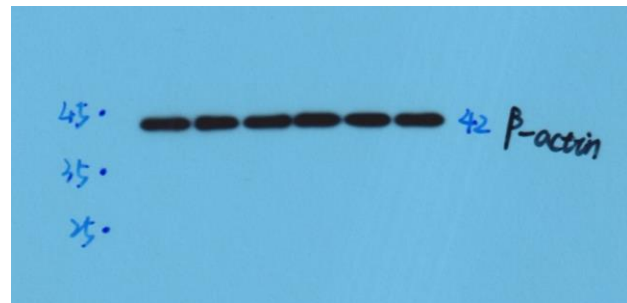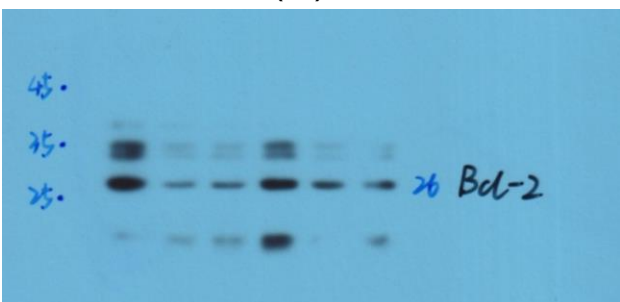

(3) √

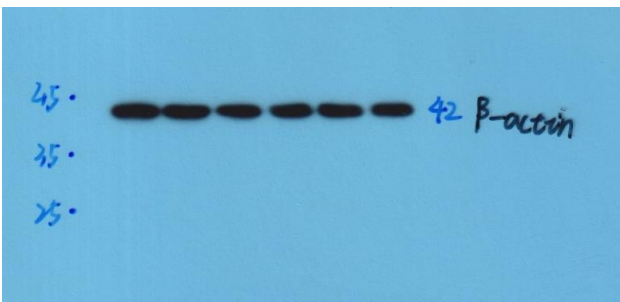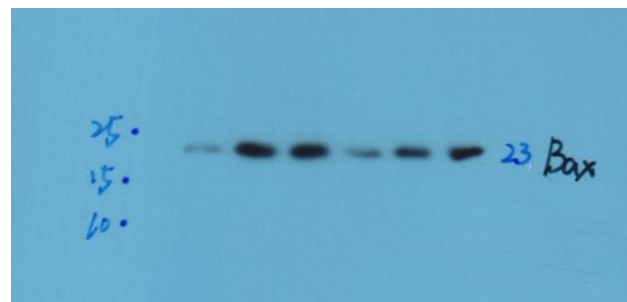

(1)

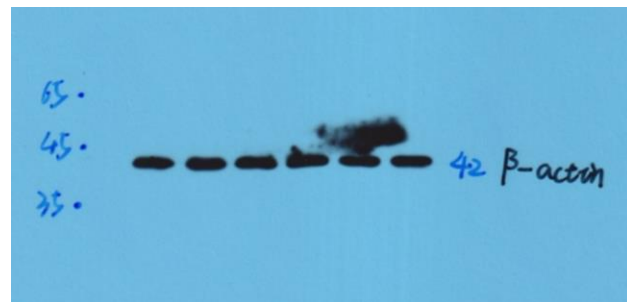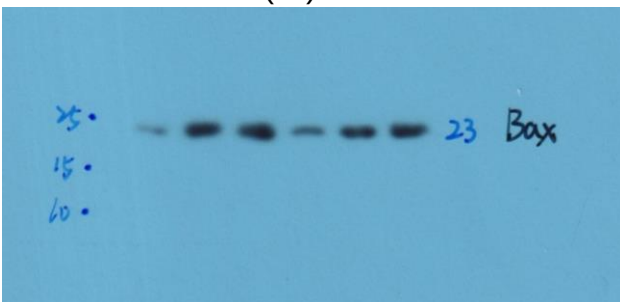

(2) √

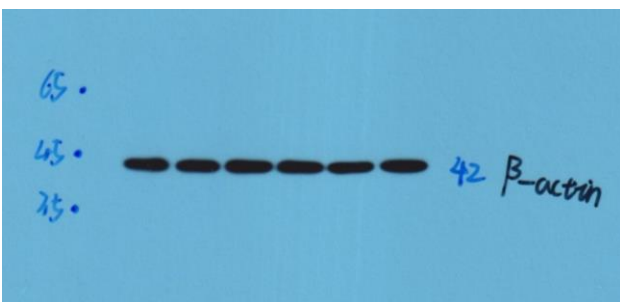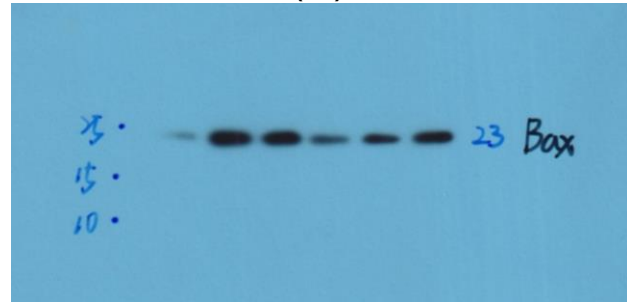

(3)

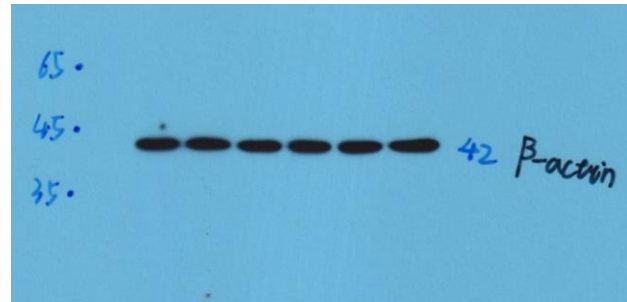

Supplement: S6 File — (PDF) [file pone.0332698.s006.pdf]
