# Supplementary material for: CLIC1 down-regulates Nrf2/HO-1 signalling pathway promoting the apoptosis and pyroptosis in OGD/R-treated HT22 cells
Source: PLoS One. 2025 Sep 18;20(9):e0332698. doi: 10.1371/journal.pone.0332698 (PMC12445528; doi:10.1371/journal.pone.0332698)
Supplement: Attachment — Submitted filename: Response to reviewers.docx [file pone.0332698.s008.docx]

**Response to Reviewers**

**Dear Editor and Reviewer**

We would like to express our sincere appreciation to the editor for overseeing the peer review process and for providing us with the valuable opportunity to revise our manuscript. We are deeply grateful for your time, effort, and guidance, which have been invaluable in improving the quality and clarity of our work.

We extend our heartfelt thanks to the reviewers for their constructive and insightful comments, which have greatly contributed to enhancing the manuscript and the impact of our research.

In response to all the suggestions, we have carefully addressed each comment and revised the manuscript accordingly. We hope that the updated version meets the journal's publication requirements. Please do not hesitate to contact us if further revisions or additional steps are required.

Thank you once again for your support and guidance throughout this process.

Sincerely,

Chuang Sun

**Journal requirements:**

Comment #1

Please ensure that your manuscript meets PLOS ONE's style requirements, including those for file naming. The PLOS ONE style templates can be found at <https://journals.plos.org/plosone/s/file?id=ba62/PLOSOne_formatting_sample_title_authors_affiliations.pdf>

We've checked the information about the manuscript and ensure that our manuscript meets PLOS ONE's style requirements, including those for file naming.

Comment #2

Thank you for stating the following financial disclosure: [This research was supported by the Liaoning Provincial Science and Technology Program (2022-MS-329), the General Program of National Natural Science Foundation of China（82071911）, and the Science and Technology Innovation Fund of Dalian（2021JJ12SN38）.]. Please state what role the funders took in the study. If the funders had no role, please state: "The funders had no role in study design, data collection and analysis, decision to publish, or preparation of the manuscript." If this statement is not correct you must amend it as needed. Please include this amended Role of Funder statement in your cover letter; we will change the online submission form on your behalf.

We have added author contributions to the cover letter, including role the funders.

**Author Contributions:** C.S. conceived and designed the study. J.X. wrote the manuscript, analyzed and interpretated the data. S.L conducted majority of the experiments and collected data. A.Z provided critical revision of the manuscript. H.C., C.Y and Y.C offered technical and material support. C.S. and J.W. offered funding acquisition. J.W. and X.Q. conducted administrative support and supervision.

Screenshot of cover letter

Comment #3

We note that your Data Availability Statement is currently as follows: [All relevant data are within the manuscript.] Please confirm at this time whether or not your submission contains all raw data required to replicate the results of your study. Authors must share the “minimal data set” for their submission. PLOS defines the minimal data set to consist of the data required to replicate all study findings reported in the article, as well as related metadata and methods (https://journals.plos.org/plosone/s/data-availability#loc-minimal-data-set-definition). For example, authors should submit the following data: - The values behind the means, standard deviations and other measures reported; - The values used to build graphs; - The points extracted from images for analysis. Authors do not need to submit their entire data set if only a portion of the data was used in the reported study. If your submission does not contain these data, please either upload them as Supporting Information files or deposit them to a stable, public repository and provide us with the relevant URLs, DOIs, or accession numbers. For a list of recommended repositories, please see https://journals.plos.org/plosone/s/recommended-repositories. If there are ethical or legal restrictions on sharing a de-identified data set, please explain them in detail (e.g., data contain potentially sensitive information, data are owned by a third-party organization, etc.) and who has imposed them (e.g., an ethics committee). Please also provide contact information for a data access committee, ethics committee, or other institutional body to which data requests may be sent. If data are owned by a third party, please indicate how others may request data access.

We supplement the data and upload them as supporting information (data 1-5). Please check the supporting information.

Comment #4

PLOS requires an ORCID iD for the corresponding author in Editorial Manager on papers submitted after December 6th, 2016. Please ensure that you have an ORCID iD and that it is validated in Editorial Manager. To do this, go to ‘Update my Information’ (in the upper left-hand corner of the main menu), and click on the Fetch/Validate link next to the ORCID field. This will take you to the ORCID site and allow you to create a new iD or authenticate a pre-existing iD in Editorial Manager.

We have added the ORCID iD of all corresponding authors (Chuang Sun) in ‘Update my Information’.

Comment #5

Please include a caption for figure 8.

We are very sorry, due to an oversight in the original manuscript, it was mislabeled as figure 7 in figure legend and has been corrected. The heat maps are Image 8.

Comment #6

Please ensure that you refer to Figure 7 in your text as, if accepted, production will need this reference to link the reader to the figure.

We are very sorry, due to an oversight in the original manuscript, it was mislabeled as figure 6 in the results part and has been corrected.

**Reviewer #1:**

This study examines the role of chloride intracellular channel 1 (CLIC1) in oxidative stress and inflammation during cerebral ischemia–reperfusion injury (CIRI) using an oxygen and glucose deprivation/reoxygenation (OGD/R) model in HT22 hippocampal neurons. The results show that CLIC1 is significantly upregulated under OGD/R conditions, leading to neuronal damage by suppressing the Nrf2/HO-1 signaling pathway. This suppression exacerbates oxidative stress, inducing apoptosis and NLRP3 inflammasome-mediated pyroptosis. Silencing CLIC1 alleviates these effects, reducing oxidative stress markers, inflammasome activity, and cell death. The findings highlight CLIC1's dual role in apoptosis and pyroptosis and suggest its potential as a therapeutic target to mitigate CIRI-induced neuronal injury by modulating the Nrf2/HO-1 pathway. I recommend publishing this manuscript with the inclusion of the following significant corrections, elaborated below, to further enhance its clarity, robustness, and scientific rigor.

**Dear Reviewer,**

We sincerely thank you for their thoughtful and comprehensive feedback on our study. We also thank you for the constructive suggestions to improve the clarity, robustness, and scientific rigor of our manuscript. We have carefully addressed each of your recommendations to enhance the quality and impact of our work.

The specific responses are as follows:

Reviewer’s Comment #1

Figures: Figures need higher resolution (300–600 dpi, per PLOS ONE guidelines) and more detailed, self-explanatory legends to improve clarity and interpretability.

We have revised all the images by updating the labeling and enhancing its resolution for better visualization. Additionally, we have rewritten the figure legends to improve clarity and ensure better interpretability of the data presented.

For Example:

Fig 3. CLIC1 upregulation accelerates oxidative stress in oxygen and glucose deprivation/reoxygenation (OGD/R)-treated HT22 cells. The activities of SOD (a), CAT (b), and GSH-Px (c) were significantly reduced in OGD/R-treated HT22 cells compared to the control group, with the restoration of these antioxidant enzyme activities upon CLIC1 silencing. (d) The LDH level was elevated in the OGD/R group and decreased following CLIC1 silencing. (e) The CCK-8 OD value was reduced in the OGD/R group and increased with CLIC1 silencing. (f-h) Real-time PCR (f) and WB (g, h) analyses showed that OGD/R treatment decreased anti-apoptotic Bcl-2 expression and increased pro-apoptotic Bax expression, with these effects reversed by CLIC1 silencing. Data are presented as mean ± SEM, with n = 3 per group. *P<0.05 versus control group; ^P<0.05 versus OGD/R group.

Reviewer’s Comment #2

Heatmaps: Gene expression and correlation heatmaps require better labeling for easier understanding.

We have reworked and revised the heatmap, adding new labels and updating the legend to improve its clarity and comprehensibility.

Fig 8. Heatmaps of gene expression and correlation analysis for molecular changes across the control, oxygen and glucose deprivation/reoxygenation (OGD/R), OGD/R+ negative control (NC), OGD/R+CLIC1 silencing, OGD/R+CLIC1 silencing+Nrf2/HO-1 inhibition, and OGD/R+CLIC1 silencing+NLRP3 activation groups. (a) Gene expression heatmap illustrates the relative expression levels of key genes (detected by real-time PCR) across all the six groups. Each row represents a specific gene, and each column represents a sample. (b) Correlation heatmap showing the Spearman correlation coefficients among the genes detected by real-time PCR. Each cell represents the correlation between two genes, with the colour gradient indicating the strength and direction of the correlation.

Reviewer’s Comment #3

Abbreviation Use: Abbreviations (e.g., NLRP3, GSDMD) should be defined at first mention and used consistently throughout the text.

We sincerely apologize for the clerical error and have corrected the relevant abbreviations throughout the manuscript. All concepts are now fully presented upon their first mention, with abbreviations consistently and accurately applied thereafter.

Reviewer’s Comment #4

Reference Formatting: The reference list does not fully adhere to journal guidelines. Full journal names (e.g., "Biomedicine & Pharmacotherapy" instead of "Biomed Pharmacother") and consistent DOI formatting are required.

We have carefully revised the reference format to align with your suggestions and the author guidelines of PLOS ONE.

For example:

Reviewer’s Comment #5

Language and Grammar: Minor grammatical issues (e.g., “to promoted apoptosis” instead of “to promote apoptosis”) and wordiness reduce readability and need correction.

As non-native English speakers, we acknowledge that the manuscript contained some language and grammatical misapplications, and we sincerely apologize for these issues. To address and resolve these problems, we have thoroughly reviewed and corrected the full text and engaged a professional agency (Charlesworth, UK) to refine the article further. This process has corrected linguistic and grammatical errors and significantly enhanced the overall readability of the manuscript.

Reviewer’s Comment #6

Novelty: The study needs to better emphasize its novel contribution, particularly the regulatory role of CLIC1 in the Nrf2/HO-1 pathway and its implications.

Thank you for highlighting the need to better emphasize the novel contribution of our study. We agree that this is a critical aspect of our work, and we have undertaken revisions to strengthen the emphasis on this point throughout the manuscript as follows:

**1. Introduction:** We have clarified the knowledge gap addressed by our study, particularly focusing on the lack of understanding of CLIC1's involvement in the regulation of the Nrf2/HO-1 pathway and its potential therapeutic implications.

“…Therefore, this study presents a novel insight into the regulatory role of CLIC1 in CIRI via Nrf2/HO-1 pathway using a cell model, emphasizing its critical involvement in oxidative stress and inflammation. We hypothesized that CLIC1 is highly expressed in OGD/R-treated HT22 cells and inhibits the Nrf2/HO-1 signalling pathway. These processes promote apoptosis and NLRP3-mediated pyroptosis. … These findings could unveil promising targets for the treatment of stroke and CIRI, ultimately improving outcomes and broadening the therapeutic options. “

**2. Discussion Section:** We firstly discussed the current stage of treatment of CIRI and its problems, and then proposed the potential of CLIC1 inhibitors for the treatment of CIRI by exploring their application in the central nervous system.

“ Currently, the primary long-term objective of CIRI therapy aims to restore blood flow, reduce secondary damage, and minimize stroke-related neuronal cell death, disability, and mortality [1]. Various pharmacological and interventional therapies have been developed to mitigate CIRI. The current treatment strategies for CIRI include pre-ischemic preconditioning, post-ischemic preconditioning, and pharmacological preconditioning. However, the complexity of CIRI and the intricate interplay among various signalling pathways present significant challenges, leading to a lack of consensus in existing research [2, 3]. Neuroprotective measures such as antioxidants and anti-inflammatory agents have shown promise in preclinical CIRI models [4]. Although there are no specific studies directly focusing on CLIC1 inhibitors for CIRI, previous studies [5-8] have suggested CLIC1 as a potential therapeutic target for central nervous system diseases, including brain tumours and neurodegenerative diseases. For example, inhibition of CLIC1 channels using specific blockers may effectively suppress microglial proliferation and mitigated the neurotoxicity induced by Aβ-treated microglial cells [8, 9]. CLIC1 was also identified as a critical protein linked to menopausal brain changes and increased glioma[6]. Meanwhile, in glioblastoma, CLIC1 modulates the tumour microenvironment, promoting cancer cell proliferation and invasion [10]. These findings demonstrate the potential of CLICI inhibitors in the treatment of CIRI.”

**3. Conclusion:** We have revised the conclusion to succinctly underscore the novelty of our findings and their potential impact on stroke treatment strategies.

“…The critical role of CLIC1 in mediating oxidative stress, inflammation, and neuronal damage highlights its potential as a therapeutic target through regulation of the Nrf2/HO-1 signalling pathway. This targeted strategy for CIRI could address the current treatment gaps and substantially advance ischemic stroke therapy. “

Reviewer’s Comment #7

Mechanistic Clarity: While the study links CLIC1 to pyroptosis/apoptosis via Nrf2/HO-1, it does not explore alternative pathways or contributing factors, such as other stress-response mechanisms.

We are grateful to the reviewer for highlighting the need to explore alternative pathways and contributing factors beyond the Nrf2/HO-1 axis in the mechanistic link between CLIC1 and pyroptosis/apoptosis. In response, we have expanded the Discussion section to address this important point. We emphasize the potential roles of other pathways, such as Wnt, NF-κB, and MAPK, and their relevance in cellular stress responses, inflammation, and apoptosis. Their potential crosstalk with CLIC1 deserves further study. We also discuss future research directions to explore the mechanistic and therapeutic implications of these findings.

**Discussion:** “While our study primarily focuses on the mechanistic association between CLIC1 and Nrf2/HO-1 in regulating apoptosis and pyroptosis, additional oxidative stress-response pathways or regulatory networks, such as Wnt, NF-κB and MAPK signalling [2, 4, 11], might also play a role in the observed phenomena. Farrerol was reported to inhibit microglia activation by anti-inflammation and anti-apoptosis, decreasing the activation of NF-κB and MAPK signalling pathways to protect against CIRI [11]. It has been shown that the activation of the classical Wnt pathway during ischemia exerts organ-protective effects, whereas its inhibition during reperfusion exacerbates injury. In contrast, the non-classical Wnt pathway contributes to injury during both ischemic and reperfusion phases. In addition, the Wnt pathway is related to apoptosis, iron death, inflammatory response, oxidative stress, neurogenesis, angiogenesis, and blood-brain barrier damage in CIRI [2]. Electroacupuncture was reported to modulate NLRP3/ASC/caspase-1-mediated cellular focal death to protect against neuronal injury in CIRI [12]. However, these pathways and innovative approaches to treatment are critical contributors to cellular stress responses, inflammation, and apoptosis, and their potential crosstalk with CLIC1 warrants further investigation. Further research is essential to develop and validate selective CLIC1 inhibitors to improve their clinical application. We will further investigate CIRI treatment through molecular docking or macromolecular channel screening drugs in the future.”

Reviewer’s Comment #8

Experimental Design:
- Control Groups: Details on control groups, including sham-treated groups, are insufficient, raising concerns about baseline comparisons.

- Sample Size: With only three replicates per group, conclusions may lack statistical robustness. A power analysis is needed.

**Control Groups:**

We sincerely thank the reviewer for highlighting the need for additional details regarding the control groups. In response, we have provided a more comprehensive description of the control groups in the Methods of the revised manuscript, particularly focusing on the sham-treated groups used as baselines for comparison. As follows:

Control group: “…The HT22 cells in the control group were inoculated in 6-well culture plates and incubated overnight for cell processing. The cells were incubated in glucose-free Hank's Balanced Salt Solution (HBSS) under 95% air and 5% CO2 at 37 ℃ for 6 h.”

Negative Control (NC) groups: “The control HT22 cells and OGD/R-induced cells were then infected with the CLIC1 silencing-negative control virus (WL117218-2, Wanleibio, China) to create the negative control (NC) groups. The viral stock was thawed at 4°C. The virus solution was prepared by accurately measuring the required volume to achieve a multiplicity of infection (MOI) of 50 and mixing it with the culture medium. The existing cell medium was replaced with the virus-containing medium. The cells were gently mixed to ensure even distribution and incubated at 37°C with 5% CO₂. After 48 h, the cells were transferred to glucose-free HBSS and underwent the OGD/R protocol described above. “

**Sample Size:**

We performed a retrospective power analysis using G*Power 3.1 software to whether the current sample size was sufficient to detect meaningful effects. The analysis was conducted for an ANOVA test with repeated measures and between factors, assuming a medium effect size (f = 0.25), a significance level (α) of 0.05, and a desired power (1-β) of 0.8.

Since each sample was measured 3 times and averaged, the minimum total sample size =78/6 = 13. So, the power analysis results indicated that a total sample size of 13 would be required to achieve the desired statistical power of 0.8. With the actual sample size of 18 used in this study, the calculated power was 0.8246, which exceeds the commonly accepted threshold of 0.8. Therefore, the sample size employed in this study is sufficient to detect statistically significant effects. In addition, many similarly designed animal or cellular experiments were statistically analyzed at 3 samples per group. For example:

**Example 1**

Zhao R, Zhou X, Zhao Z, Liu W, Lv M, Zhang Z, Wang C, Li T, Yang Z, Wan Q, Xu R, Cui Y. Farrerol Alleviates Cerebral Ischemia-Reperfusion Injury by Promoting Neuronal Survival and Reducing Neuroinflammation. Mol Neurobiol. 2024 Sep;61(9):7239-7255. DOI: [10.1007/s12035-024-04031-9](https://doi.org/10.1007/s12035-024-04031-9). Epub 2024 Feb 20. PMID: 38376762.

**Fig. 4** FAR decreases neuronal death by activating CREB pathway and reducing oxidative stress. (**a**, **b**) Representative immunoblot analysis (**a**) and quantitative analysis (**b**) of neuroprotective signal protein in cortical neurons subjected to OGD2h/R6h treatment. (**c**, **d**) Representative immunoblot analysis (**c**) and quantification analy- sis (**d**) of CREB expression in tMCAO brain, *n*=3 mice per group. The data are means±SD, for all panels: **P*<0.05, ***P*<0.01, ****P*<0.001, n.s., no significance. One-way (**d**, **i**) or two-way (**b**, **e**, **f**, **g**) ANOVA analysis was performed followed by Tukey’s HSD test.

**Example 2**

Barbieri F, Würth R, Pattarozzi A, Verduci I, Mazzola C, Cattaneo MG, Tonelli M, Solari A, Bajetto A, Daga A, Vicentini LM, Mazzanti M, Florio T. Inhibition of Chloride Intracellular Channel 1 (CLIC1) as Biguanide Class-Effect to Impair Human Glioblastoma Stem Cell Viability. Front Pharmacol. 2018 Aug 21;9:899. DOI: [10.3389/fphar.2018.00899](https://doi.org/10.3389/fphar.2018.00899). PMID: 30186163; PMCID: PMC6110922.

Reviewer’s Comment #9

Pathway Validation: The evidence for Nrf2/HO-1 inhibition or silencing requires stronger validation, such as co-immunoprecipitation or experiments using pathway-specific inhibitors.

We appreciate the reviewer's suggestion to incorporate stronger validation approaches, such as co-immunoprecipitation or pathway-specific inhibitors. While these techniques are indeed valuable, due to current technical and resource constraints, additional experiments could not be conducted at this stage. However, our study follows a widely accepted research approach, as demonstrated in previous studies (PMID: 26780453, 34102010, 31231583，and 33453713), where direct measurement of Nrf2/HO-1 signaling molecules after pathway interference effectively validated pathway regulation. These references, as well as our current findings, strongly support the involvement of the Nrf2/HO-1 pathway in neuronal injury. We plan to incorporate stronger validation methods, such as pathway-specific inhibitors or co-immunoprecipitation, in future studies to further enhance the evidence for these mechanisms." Based on the literature review:

1. PKCε and Nrf2/HO-1 Regulation: Previous work (PMID: 26780453[13]，34102010[14]) explored whether PKCε could regulate the Nrf2/HO-1 pathway in the context of nerve injury. In those studies, Nrf2/HO-1 signaling molecules were directly measured after PKCε interference, confirming that PKCε modulates this pathway. This aligns well with the framework of our study, where we investigated the role of CLIC1 and its downstream effects on Nrf2/HO-1 signaling.
2. HDAC6 and Nrf2/HO-1 Inhibition: In the literature (PMID: 31231583[15]), HDAC6 was shown to inhibit the Nrf2/HO-1 signaling pathway in the brain tissue of MCAO mice. Similarly, Nrf2/HO-1 expression levels were directly assessed following HDAC6 interference to confirm its regulatory role. The methodology used here provides a strong precedent for our approach and reinforces the validity of measuring pathway-specific molecular alterations to establish regulation.
3. miR-133a-3p/BACH1 Axis and Nrf2/HO-1 Modulation: Another related study (PMID: 33453713[16]) established a cellular oxidative stress injury model to explore whether the miR-133a-3p/BACH1 axis could regulate the Nrf2/HO-1 pathway. By manipulating miR-133a-3p or BACH1 expression levels and measuring downstream Nrf2/HO-1 signaling, the study demonstrated a mechanistic link. This further highlights the consistency of such molecular approaches with our study, where we investigated whether CLIC1 regulates Nrf2/HO-1 signaling during CIRI.

Reviewer’s Comment #10

Methods: Experimental details, including validation of viral silencing and OGD/R treatment conditions, are inadequately described, which may hinder reproducibility.

Thank you for pointing out the need to provide more detailed experimental descriptions to ensure reproducibility. We have carefully revised and expanded the Methods section to include additional details on the validation of viral silencing and the OGD/R treatment conditions. The following updates and refinements have been made to the manuscript:

**Viral silencing:** “The control HT22 cells and OGD/R-induced cells were then infected with either the CLIC1 silencing virus (WL117218-1, Wanleibio, China) to create the CLIC1 silencing groups. The viral stock was thawed at 4°C. The virus solution was prepared by accurately measuring the required volume to achieve a multiplicity of infection (MOI) of 50 and mixing it with the culture medium. The existing cell medium was replaced with the virus-containing medium. The cells were gently mixed to ensure even distribution and incubated at 37°C with 5% CO₂. After 48 h, the cells were transferred to glucose-free HBSS and underwent the OGD/R protocol described above. “

**OGD/R treatment conditions:** “To induce OGD/R, HT22 cells were seeded in 6-well culture plates and cultured overnight to ensure proper adherence. Hypoxia was induced by replacing the culture medium with glucose-free Hank's Balanced Salt Solution (HBSS) and incubating the cells in an anoxic chamber maintained at 37°C with a gas mixture of 95% N₂ and 5% CO₂ for 6 hours. Then in reperfusion phase following hypoxic treatment, the glucose-free HBSS was replaced with normal DMEM medium (supplemented with glucose and 10% FBS), and the cells were cultured under air conditions (37°C, 21% O₂, 5% CO₂) for 24 hours. Cells were subsequently used for further experiments.”

**Reviewer #2:**

In this manuscript, the authors use a well-established model (OGD/R-treated HT22 cells) and robust methodologies, including real-time PCR, western blotting, flow cytometry, and ELISA, to investigate apoptosis and pyroptosis. The study links CLIC1 overexpression with oxidative stress-induced apoptosis and NLRP3-mediated pyroptosis, providing mechanistic evidence via modulation of the Nrf2/HO-1 pathway. To enhance the study's rigor, several improvements could be made:

**Dear Reviewer,**

We sincerely thank you for their thoughtful and comprehensive feedback on our study. We also thank you for the constructive suggestions to improve the clarity, robustness, and scientific rigor of our manuscript. We have carefully addressed each of your recommendations to enhance the quality and impact of our work.

The specific responses are as follows:

Reviewer’s Comment #1

Lack of In Vivo Validation: While the cell model findings are significant, they may not fully translate to in vivo systems. Including animal studies would strengthen the conclusions. Please add in vivo experimental verification if conditions permit.

We sincerely thank the reviewer for their valuable suggestions, which are critical to enhancing the rigor and depth of our research. As requested, additional relevant experiments have been conducted; however, the comprehensive methods and results of these animal studies have been described in detail in a separate article submitted to another journal. To provide clarity for this manuscript, we outline below the key aspects of the methods and results from the animal study:

**Methods:** We used 24 healthy, 5-week-old male Sprague–Dawley rats, which were acclimated for 2 weeks in a controlled environment. They were divided into sham, MCAO/R, NC, and CLIC1-GS groups (6 rats/group). Under sevoflurane anesthesia, rats underwent surgery where the left carotid arteries were isolated, and blood flow to the left middle cerebral artery was blocked using a nylon thread. Reperfusion was induced 2 hours later by removing the thread and suturing the incision. Sham rats underwent similar procedures without vascular ligation or thread insertion. After 24 hours, the Longa neurological scoring method was used to confirm the success of the MCAO/R model.

**Results:** The expression of CLIC1, caspase-3, NLRP3, GSDMD, IL-1β, IL-18, ROS, and other related parameters was higher, whereas that of Nrf2/HO-1 was lower in the middle cerebral artery occlusion/reperfusion (MCAO/R) group than in the sham group. Additionally, hippocampal cell edema and oxidative stress were aggravated, and the apoptosis rate increased in the MCAO/R group. CLIC1 silencing alleviated the oxidative stress. Nrf2 and HO-1 expression levels were higher in the CLIC1-GS group than in the non-control (NC) group.

We appreciate the reviewer’s understanding that the extensive details regarding the design and findings of the animal study are reserved for the separate article but believe this brief overview aligns with the context of the current manuscript. Once again, we thank the reviewer for their insightful input, which has helped to strengthen the scientific foundation of our work.

Reviewer’s Comment #2

Some sentences are overly complex, leading to occasional ambiguity (e.g., in the introduction and discussion).

To address this concern, we have carefully re-edited and reorganized the sentences across the manuscript, with particular attention to these sections. Our revision process involved:

1. Simplifying overly complex sentences by breaking them into shorter, more concise statements while ensuring that the scientific content remains accurate and complete.

2.Reorganizing the structure of paragraphs to improve the logical flow of ideas and enhance their clarity.

3.Revising specific terms or phrasing that could cause ambiguity, replacing them with clearer and more precise expressions.

By implementing these changes, we hope to enhance the article's readability and comprehensibility while minimizing potential ambiguity.

Reviewer’s Comment #3

Several grammatical errors and awkward sentence structures are present. For example: "And this process led to an exacerbation of apoptosis..." → Avoid starting sentences with "And." "Upon CLIC1 silencing, all the mentioned indicators above exhibited an inverse trend..." → Simplify to: "Silencing CLIC1 reversed these effects." Repetition: The phrase "CLIC1 silencing" is overused. Vary terminology to improve readability.

We sincerely thank the reviewers for identifying the language issues in our manuscript. As non-native English speakers, we acknowledge and apologize for these errors, and we humbly accept the criticism. To address these concerns, we have thoroughly reorganized and meticulously reviewed the entire text. Additionally, we engaged a professional language editing agency (Charles, UK) to comprehensively polish the manuscript, focusing on correcting inappropriate expressions, grammatical errors, and repetitive wording. We are confident that these improvements have significantly enhanced the linguistic quality and readability of the manuscript.

Reviewer’s Comment #4

In the discussion section, while the discussion highlights the importance of the findings, it occasionally reiterates results rather than expanding on their broader implications.

We sincerely thank the reviewer for their insightful feedback regarding the discussion section. In response, we have carefully revised the discussion section to minimize redundancy and provide a more in-depth exploration of the study’s significance. Specifically, we have expanded on the potential applications of CLIC1 inhibitors and emphasized the therapeutic prospects of targeting CLIC1 and related pathways in the treatment of CIRI.

Below, we outline some of the key refinements made to the **Discussion**:

**P1**… Mechanistically, we demonstrated that CLIC1 acts as a negative regulator of the Nrf2/HO-1 signalling pathway, a critical antioxidant defence mechanism. By inhibiting Nrf2/HO-1, CLIC1 amplified redox imbalance, oxidative stress, apoptosis, and pyroptosis. Furthermore, silencing CLIC1 enhanced Nrf2/HO-1 activity, alleviating neuronal injury. These findings propose CLIC1 as a novel molecular target for mitigating oxidative stress-induced neuronal damage and the inflammatory response in CIRI.

**P3**…In our study, the observed changes in apoptotic markers, including the pro-apoptotic Bax and anti-apoptotic Bcl-2 [17], further highlight the role of CLIC1 in regulating apoptosis in OGD/R-treated HT22 cells. The upregulation of Bax and downregulation of Bcl-2 are known to activate the caspase cascade, promoting proteolysis and neuronal apoptosis [18-20]. Prior research has shown that oxidative stress-induced apoptosis in CIRI can be alleviated by modulating the Bcl-2/Bax signalling pathway, which underscores the potential of targeting CLIC1 to mitigate neuronal damage [21]. These findings suggest that CLIC1 may contribute to CIRI by fostering a redox imbalance and promoting apoptosis, positioning it as a potential therapeutic target.

**P4**…These interconnected roles of NLRP3 inflammasome activation and CLIC1 regulation highlight the broader implications of inflammatory and ionic homeostasis pathways in CIRI. Therapeutic interventions targeting these mechanisms could provide multifaceted protection, curbing inflammation and stabilizing cellular environments, suggesting potential avenues for more effective treatment strategies in ischemic stroke management.

**P5**…Together, these cell death mechanisms play distinct yet overlapping roles in both eliminating damaged cells and activating immune responses to CIRI. However, their relative contributions in CIRI depend on variables such as the specific experimental model used, the severity and duration of ischemia, and the extent of reperfusion injury. Considering this complexity, therapeutic approaches that integrate both anti-inflammatory and antioxidant strategies hold great potential for managing CIRI.

**P6**…Currently, the primary long-term objective of CIRI therapy aims to restore blood flow, reduce secondary damage, and minimize stroke-related neuronal cell death, disability, and mortality [1]. Various pharmacological and interventional therapies have been developed to mitigate CIRI. The current treatment strategies for CIRI include pre-ischemic preconditioning, post-ischemic preconditioning, and pharmacological preconditioning. However, the complexity of CIRI and the intricate interplay among various signalling pathways present significant challenges, leading to a lack of consensus in existing research [2, 3]. Neuroprotective measures such as antioxidants and anti-inflammatory agents have shown promise in preclinical CIRI models [4]. Although there are no specific studies directly focusing on CLIC1 inhibitors for CIRI, previous studies [5-8] have suggested CLIC1 as a potential therapeutic target for central nervous system diseases, including brain tumours and neurodegenerative diseases. For example, inhibition of CLIC1 channels using specific blockers may effectively suppress microglial proliferation and mitigated the neurotoxicity induced by Aβ-treated microglial cells [8, 9]. CLIC1 was also identified as a critical protein linked to menopausal brain changes and increased glioma[6]. Meanwhile, in glioblastoma, CLIC1 modulates the tumour microenvironment, promoting cancer cell proliferation and invasion [10]. These findings demonstrate the potential of CLICI inhibitors in the treatment of CIRI.

These revisions aim to provide a more comprehensive and forward-looking analysis of the study’s findings. We hope these changes address your concerns and enhance the clarity and depth of the discussion section. Again, we are grateful for your valuable feedback, which has significantly contributed to improving the manuscript.

**References**

1.	Li M, Tang H, Li Z, Tang W. Emerging Treatment Strategies for Cerebral Ischemia-Reperfusion Injury. Neuroscience. 2022;507:112-124.doi:10.1016/j.neuroscience.2022.10.020

2.	Zhang M, Liu Q, Meng H, Duan H, Liu X, Wu J, et al. Ischemia-reperfusion injury: molecular mechanisms and therapeutic targets. Signal Transduct Target Ther. 2024;9(1):12.doi:10.1038/s41392-023-01688-x

3.	Pell V R, Spiroski A M, Mulvey J, Burger N, Costa A S H, Logan A, et al. Ischemic preconditioning protects against cardiac ischemia reperfusion injury without affecting succinate accumulation or oxidation. J Mol Cell Cardiol. 2018;123:88-91.doi:10.1016/j.yjmcc.2018.08.010

4.	Xu Q, Cheung R T F. Melatonin at repeated doses alleviates hyperglycemia-exacerbated cerebral ischemia-reperfusion injury at 72 h via anti-inflammation and anti-apoptosis. IBRO Neurosci Rep. 2024;16:418-427.doi:10.1016/j.ibneur.2024.03.003

5.	Chae B J, Lee K S, Hwang I, Yu J W. Extracellular Acidification Augments NLRP3-Mediated Inflammasome Signaling in Macrophages. Immune Netw. 2023;23(3):e23.doi:10.4110/in.2023.23.e23

6.	Liu C, Zhang W, Xu G, Zhang D, Zhang C, Qiao S, et al. Deep multilayer brain omics identifies the potential involvement of menopause molecular networks in Gliomas' disease progression. FASEB J. 2022;36(10):e22570.doi:10.1096/fj.202200427RR

7.	Domingo-Fernandez R, Coll R C, Kearney J, Breit S, O'Neill L A J. The intracellular chloride channel proteins CLIC1 and CLIC4 induce IL-1beta transcription and activate the NLRP3 inflammasome. Journal of Biological Chemistry. 2017;292(29):12077-12087.doi:10.1074/jbc.M117.797126

8.	Luo L, Song S, Ezenwukwa C C, Jalali S, Sun B, Sun D. Ion channels and transporters in microglial function in physiology and brain diseases. Neurochem Int. 2021;142:104925.doi:10.1016/j.neuint.2020.104925

9.	Kettenmann H, Hanisch U K, Noda M, Verkhratsky A. Physiology of microglia. Physiol Rev. 2011;91(2):461-553.doi:10.1152/physrev.00011.2010

10.	Peretti M, Raciti F M, Carlini V, Verduci I, Sertic S, Barozzi S, et al. Mutual Influence of ROS, pH, and CLIC1 Membrane Protein in the Regulation of G(1)-S Phase Progression in Human Glioblastoma Stem Cells. Mol Cancer Ther. 2018;17(11):2451-2461.doi:10.1158/1535-7163.MCT-17-1223

11.	Zhao R, Zhou X, Zhao Z, Liu W, Lv M, Zhang Z, et al. Farrerol Alleviates Cerebral Ischemia-Reperfusion Injury by Promoting Neuronal Survival and Reducing Neuroinflammation. Molecular Neurobiology. 2024;61(9):7239-7255.doi:10.1007/s12035-024-04031-9

12.	Tang B, Li Y, Xu X, Du G, Wang H. Electroacupuncture Ameliorates Neuronal Injury by NLRP3/ASC/Caspase-1 Mediated Pyroptosis in Cerebral Ischemia-Reperfusion. Molecular Neurobiology. 2024;61(4):2357-2366.doi:10.1007/s12035-023-03712-1

13.	Guo C, Wang S, Duan J, Jia N, Zhu Y, Ding Y, et al. Protocatechualdehyde Protects Against Cerebral Ischemia-Reperfusion-Induced Oxidative Injury Via Protein Kinase Cepsilon/Nrf2/HO-1 Pathway. Molecular Neurobiology. 2017;54(2):833-845.doi:10.1007/s12035-016-9690-z

14.	Xue Z, Zhao K, Sun Z, Wu C, Yu B, Kong D, et al. Isorhapontigenin ameliorates cerebral ischemia/reperfusion injury via modulating Kinase Cepsilon/Nrf2/HO-1 signaling pathway. Brain and Behavior. 2021;11(7):e02143.doi:10.1002/brb3.2143

15.	Li J, Zhao Y, Shi J, Ren Z, Chen F, Tang W. Histone deacetylase 6 interference protects mice against experimental stroke-induced brain injury via activating Nrf2/HO-1 pathway. Anim Cells Syst (Seoul). 2019;23(3):192-199.doi:10.1080/19768354.2019.1601132

16.	Guo H, Wang Y, Jia W, Liu L. MiR-133a-3p relieves the oxidative stress induced trophoblast cell apoptosis through the BACH1/Nrf2/HO-1 signaling pathway. Physiol Res. 2021;70(1):67-78.doi:10.33549/physiolres.934506

17.	Liu J F, Su G, Chen L X, Zhou J P, Gao J, Zhang J J, et al. Irisin Attenuates Apoptosis Following Ischemia-Reperfusion Injury Through Improved Mitochondria Dynamics and ROS Suppression Mediated Through the PI3K/Akt/mTOR Axis. Molecular Neurobiology. 2023;60(8):4261-4272.doi:10.1007/s12035-023-03336-5

18.	Valko M, Leibfritz D, Moncol J, Cronin M T, Mazur M, Telser J. Free radicals and antioxidants in normal physiological functions and human disease. International Journal of Biochemistry & Cell Biology. 2007;39(1):44-84.doi:10.1016/j.biocel.2006.07.001

19.	Kluck R M, Bossy-Wetzel E, Green D R, Newmeyer D D. The release of cytochrome c from mitochondria: a primary site for Bcl-2 regulation of apoptosis. Science. 1997;275(5303):1132-1136.doi:10.1126/science.275.5303.1132

20.	Sun X, Cui X. Isorhapontigenin alleviates cerebral ischemia/reperfusion injuries in rats and modulated the PI3K/Akt signaling pathway. Naunyn-Schmiedeberg's Archives of Pharmacology. 2020;393(9):1753-1760.doi:10.1007/s00210-019-01794-0

21.	Zhou S, Gao X, Chen C, Zhang J, Zhang Y, Zhang L, et al. Porcine cardiac blood - Salvia miltiorrhiza root alleviates cerebral ischemia reperfusion injury by inhibiting oxidative stress induced apoptosis through PI3K/AKT/Bcl-2/Bax signaling pathway. Journal of Ethnopharmacology. 2023;316:116698.doi:10.1016/j.jep.2023.116698
