# Supplementary material for: CLIC1 down-regulates Nrf2/HO-1 signalling pathway promoting the apoptosis and pyroptosis in OGD/R-treated HT22 cells
Source: PLoS One. 2025 Sep 18;20(9):e0332698. doi: 10.1371/journal.pone.0332698 (PMC12445528; doi:10.1371/journal.pone.0332698)
Supplement: Attachment — Submitted filename: Response_to_Reviewers_auresp_2.docx [file pone.0332698.s009.docx]

Dear Editor,

Thank you for providing us with the opportunity to revise our manuscript and for the valuable feedback from the reviewers.

We sincerely apologize for the oversight in updating our references and for not paying close attention to the status of cited literature during the long period of writing the article. This has been an important learning experience, and we will ensure greater diligence in future writing and reference management. We are grateful for your constructive guidance, which has benefited us greatly.

We have carefully reviewed each reference and made necessary adjustments. The added references have been incorporated into the revised manuscript.

We hope these revisions improve the scientific rigor and clarity of the manuscript. Should you require any further clarification or changes, please feel free to contact us.

Thank you again for your time, support, and guidance.

Sincerely,
Chuang Sun

**Journal Requirements:**
Please review your reference list to ensure that it is complete and correct.

If you have cited papers that have been retracted, please include the rationale for doing so in the manuscript text, or remove these references and replace them with relevant current references.

Any changes to the reference list should be mentioned in the rebuttal letter that accompanies your revised manuscript.

If you need to cite a retracted article, indicate the article’s retracted status in the References list and also include a citation and full reference for the retraction notice.

**Responses:**

We have carefully reviewed each reference and made necessary adjustments.

To ensure clarity, the following actions were taken with the updated references:

1. Replaced outdated references and removed any unrecorded references.
2. Specifically addressed articles flagged under early warnings or journals excluded from recognized indexes (e.g., SCI).
3. Revised the content of related articles to align with recent findings.

**In the updated reference list:**

1. **Unrecorded references** (not included in recognized indexes or excluded from SCI) have been marked in blue.
2. **Older literature** (published before 2015) has been labeled in yellow.
3. **Chinese Academy of Sciences early warning journals** have been labeled in green.
4. **Red labeling** indicates the specific action taken for each revised literature (whether replaced, updated, or removed).

**References (Original)**

1.	Long Y, Liu S, Wan J, Zhang Y, Li D, Yu S, et al. Brain targeted borneol-baicalin liposome improves blood-brain barrier integrity after cerebral ischemia-reperfusion injury via inhibiting HIF-1alpha/VEGF/eNOS/NO signal pathway. Biomedicine & Pharmacotherapy. 2023;160:114240.doi:10.1016/j.biopha.2023.114240

2.	Yang Y, He B, Zhang X, Yang R, Xia X, Chen L, et al. Geraniin Protects against Cerebral Ischemia/Reperfusion Injury by Suppressing Oxidative Stress and Neuronal Apoptosis via Regulation of the Nrf2/HO-1 Pathway. Oxidative Medicine and Cellular Longevity. 2022;2022:2152746.doi:10.1155/2022/2152746 Deleted and replaced by other articles.

3.	Wang Z, Li Y, Ye Y, Zhu H, Zhang J, Wang H, et al. NLRP3 inflammasome deficiency attenuates cerebral ischemia-reperfusion injury by inhibiting ferroptosis. Brain Research Bulletin. 2023;193:37-46.doi:10.1016/j.brainresbull.2022.11.016

4.	Liao S, Apaijai N, Chattipakorn N, Chattipakorn S C. The possible roles of necroptosis during cerebral ischemia and ischemia / reperfusion injury. Archives of Biochemistry and Biophysics. 2020;695:108629.doi:10.1016/j.abb.2020.108629

5.	Coimbra-Costa D, Alva N, Duran M, Carbonell T, Rama R. Oxidative stress and apoptosis after acute respiratory hypoxia and reoxygenation in rat brain. Redox Biology. 2017;12:216-225.doi:10.1016/j.redox.2017.02.014

6.	Shi J, Gao W, Shao F. Pyroptosis: Gasdermin-Mediated Programmed Necrotic Cell Death. Trends in Biochemical Sciences. 2017;42(4):245-254.doi:10.1016/j.tibs.2016.10.004

7.	Xiao L, Dai Z, Tang W, Liu C, Tang B. Astragaloside IV Alleviates Cerebral Ischemia-Reperfusion Injury through NLRP3 Inflammasome-Mediated Pyroptosis Inhibition via Activating Nrf2. Oxidative Medicine and Cellular Longevity. 2021;2021:9925561.doi:10.1155/2021/9925561 Deleted and replaced by another article.

8.	Zhang J Y, Zhou B, Sun R Y, Ai Y L, Cheng K, Li F N, et al. The metabolite alpha-KG induces GSDMC-dependent pyroptosis through death receptor 6-activated caspase-8. Cell Research. 2021;31(9):980-997.doi:10.1038/s41422-021-00506-9

9.	Lamkanfi M, Dixit V M. Mechanisms and functions of inflammasomes. Cell. 2014;157(5):1013-1022.doi:10.1016/j.cell.2014.04.007 Deleted.

10.	Frank D, Vince J E. Pyroptosis versus necroptosis: similarities, differences, and crosstalk. Cell Death & Differentiation. 2019;26(1):99-114.doi:10.1038/s41418-018-0212-6

11.	Heneka M T, McManus R M, Latz E. Inflammasome signalling in brain function and neurodegenerative disease. Nature Reviews Neuroscience. 2018;19(10):610-621.doi:10.1038/s41583-018-0055-7

12.	Shi S, Zhang C, Liu J. TIMP2 facilitates CIRI through activating NLRP3-mediated pyroptosis. Aging (Albany New York). 2023;15(9):3635-3643.doi:10.18632/aging.204696

13.	Fink S L, Cookson B T. Caspase-1-dependent pore formation during pyroptosis leads to osmotic lysis of infected host macrophages. Cellular Microbiology. 2006;8(11):1812-1825.doi:10.1111/j.1462-5822.2006.00751.x Deleted and replaced by another article.

14.	Wallach D, Kang T B, Dillon C P, Green D R. Programmed necrosis in inflammation: Toward identification of the effector molecules. Science. 2016;352(6281):aaf2154.doi:10.1126/science.aaf2154

15.	Jiao-Yan Y, Qing-Qing L, Xi L, Mei Z, Ting S, Na H, et al. Oxymatrine improves blood-brain barrier integrity after cerebral ischemia-reperfusion injury by downregulating CAV1 and MMP9 expression. Phytomedicine. 2021;84:153505.doi:10.1016/j.phymed.2021.153505

16.	Uivarosan D, Bungau S, Tit D M, Moisa C, Fratila O, Rus M, et al. Financial Burden of Stroke Reflected in a Pilot Center for the Implementation of Thrombolysis. Medicina (Kaunas). 2020;56(2).doi:10.3390/medicina56020054

17.	Ozaki S, Mikami K, Kunieda T, Tanaka J. Chloride Intracellular Channel Proteins (CLICs) and Malignant Tumor Progression: A Focus on the Preventive Role of CLIC2 in Invasion and Metastasis. Cancers (Basel). 2022;14(19).doi:10.3390/cancers14194890 Deleted and replaced by another article.

18.	Lu D, Le Y, Ding J, Dou X, Mao W, Zhu J. CLIC1 Inhibition Protects Against Cellular Senescence and Endothelial Dysfunction Via the Nrf2/HO-1 Pathway. Cell Biochem Biophys. 2021;79(2):239-252.doi:10.1007/s12013-020-00959-6

19.	Domingo-Fernandez R, Coll R C, Kearney J, Breit S, O'Neill L A J. The intracellular chloride channel proteins CLIC1 and CLIC4 induce IL-1beta transcription and activate the NLRP3 inflammasome. Journal of Biological Chemistry. 2017;292(29):12077-12087.doi:10.1074/jbc.M117.797126

20.	Liu B, Billington C K, Henry A P, Bhaker S K, Kheirallah A K, Swan C, et al. Chloride intracellular channel 1 (CLIC1) contributes to modulation of cyclic AMP-activated whole-cell chloride currents in human bronchial epithelial cells. Physiological Reports. 2018;6(2).doi:10.14814/phy2.13508

21.	Cianci F, Verduci I. Transmembrane Chloride Intracellular Channel 1 (tmCLIC1) as a Potential Biomarker for Personalized Medicine. Journal of Personalized Medicine. 2021;11(7).doi:10.3390/jpm11070635 Deleted.

22.	Dong W, Qiu C, Gong D, Jiang X, Liu W, Liu W, et al. Proteomics and bioinformatics approaches for the identification of plasma biomarkers to detect Parkinson's disease. Experimental and Therapeutic Medicine. 2019;18(4):2833-2842.doi:10.3892/etm.2019.7888 Deleted.

23.	Milton R H, Abeti R, Averaimo S, DeBiasi S, Vitellaro L, Jiang L, et al. CLIC1 function is required for beta-amyloid-induced generation of reactive oxygen species by microglia. Journal of Neuroscience. 2008;28(45):11488-11499.doi:10.1523/JNEUROSCI.2431-08.2008 Deleted.

24.	Djuric U, Lam K H B, Kao J, Batruch I, Jevtic S, Papaioannou M D, et al. Defining Protein Pattern Differences Among Molecular Subtypes of Diffuse Gliomas Using Mass Spectrometry. Molecular & Cellular Proteomics. 2019;18(10):2029-2043.doi:10.1074/mcp.RA119.001521

25.	Zhang Q, Wang J, Zhang H, Zeng T. Dihydromyricetin inhibits oxidative stress and apoptosis in oxygen and glucose deprivation/reoxygenation‑induced HT22 cells by activating the Nrf2/HO‑1 pathway. Molecular Medicine Reports. 2021;23(6).doi:10.3892/mmr.2021.12036

26.	Wang L, Zhang X, Xiong X, Zhu H, Chen R, Zhang S, et al. Nrf2 Regulates Oxidative Stress and Its Role in Cerebral Ischemic Stroke. Antioxidants (Basel). 2022;11(12).doi:10.3390/antiox11122377

27.	Zhu H, Jia Z, Misra B R, Zhang L, Cao Z, Yamamoto M, et al. Nuclear factor E2-related factor 2-dependent myocardiac cytoprotection against oxidative and electrophilic stress. Cardiovascular Toxicology. 2008;8(2):71-85.doi:10.1007/s12012-008-9016-0 Deleted.

28.	Swanson K V, Deng M, Ting J P. The NLRP3 inflammasome: molecular activation and regulation to therapeutics. Nature Reviews Immunology. 2019;19(8):477-489.doi:10.1038/s41577-019-0165-0

29.	Ulmasov B, Bruno J, Woost P G, Edwards J C. Tissue and subcellular distribution of CLIC1. BMC Cell Biology. 2007;8:8.doi:10.1186/1471-2121-8-8 Deleted and replaced by another article.

30.	Francisco M A, Wanggou S, Fan J J, Dong W, Chen X, Momin A, et al. Chloride intracellular channel 1 cooperates with potassium channel EAG2 to promote medulloblastoma growth. Journal of Experimental Medicine. 2020;217(5).doi:10.1084/jem.20190971

31.	Xu M S, Yin L M, Cheng A F, Zhang Y J, Zhang D, Tao M M, et al. Cerebral Ischemia-Reperfusion Is Associated With Upregulation of Cofilin-1 in the Motor Cortex. Frontiers in Cell and Developmental Biology. 2021;9:634347.doi:10.3389/fcell.2021.634347

32.	Araujo J A, Zhang M, Yin F. Heme oxygenase-1, oxidation, inflammation, and atherosclerosis. Frontiers in Pharmacology. 2012;3:119.doi:10.3389/fphar.2012.00119 Deleted.

33.	Coll R C, Schroder K, Pelegrin P. NLRP3 and pyroptosis blockers for treating inflammatory diseases. Trends in Pharmacological Sciences. 2022;43(8):653-668.doi:10.1016/j.tips.2022.04.003

34.	Liu J F, Su G, Chen L X, Zhou J P, Gao J, Zhang J J, et al. Irisin Attenuates Apoptosis Following Ischemia-Reperfusion Injury Through Improved Mitochondria Dynamics and ROS Suppression Mediated Through the PI3K/Akt/mTOR Axis. Molecular Neurobiology. 2023;60(8):4261-4272.doi:10.1007/s12035-023-03336-5

35.	Valko M, Leibfritz D, Moncol J, Cronin M T, Mazur M, Telser J. Free radicals and antioxidants in normal physiological functions and human disease. International Journal of Biochemistry & Cell Biology. 2007;39(1):44-84.doi:10.1016/j.biocel.2006.07.001 Deleted.

36.	Kluck R M, Bossy-Wetzel E, Green D R, Newmeyer D D. The release of cytochrome c from mitochondria: a primary site for Bcl-2 regulation of apoptosis. Science. 1997;275(5303):1132-1136.doi:10.1126/science.275.5303.1132 Deleted.

37.	Sun X, Cui X. Isorhapontigenin alleviates cerebral ischemia/reperfusion injuries in rats and modulated the PI3K/Akt signaling pathway. Naunyn-Schmiedeberg's Archives of Pharmacology. 2020;393(9):1753-1760.doi:10.1007/s00210-019-01794-0

38.	Zhou S, Gao X, Chen C, Zhang J, Zhang Y, Zhang L, et al. Porcine cardiac blood - Salvia miltiorrhiza root alleviates cerebral ischemia reperfusion injury by inhibiting oxidative stress induced apoptosis through PI3K/AKT/Bcl-2/Bax signaling pathway. Journal of Ethnopharmacology. 2023;316:116698.doi:10.1016/j.jep.2023.116698

39.	Carlini V, Verduci I, Cianci F, Cannavale G, Fenoglio C, Galimberti D, et al. CLIC1 Protein Accumulates in Circulating Monocyte Membrane during Neurodegeneration. International Journal of Molecular Sciences. 2020;21(4).doi:10.3390/ijms21041484

40.	Place D E, Kanneganti T D. Recent advances in inflammasome biology. Current Opinion in Immunology. 2018;50:32-38.doi:10.1016/j.coi.2017.10.011

41.	Luo J F, Shen X Y, Lio C K, Dai Y, Cheng C S, Liu J X, et al. Activation of Nrf2/HO-1 Pathway by Nardochinoid C Inhibits Inflammation and Oxidative Stress in Lipopolysaccharide-Stimulated Macrophages. Frontiers in Pharmacology. 2018;9:911.doi:10.3389/fphar.2018.00911

42.	Orekhov A N, Nikiforov N G, Omelchenko A V, Sinyov V V, Sobenin I A, Vinokurov A Y, et al. The Role of Mitochondrial Mutations in Chronification of Inflammation: Hypothesis and Overview of Own Data. Life (Basel). 2022;12(8).doi:10.3390/life12081153

43.	Bellut M, Papp L, Bieber M, Kraft P, Stoll G, Schuhmann M K. NLPR3 inflammasome inhibition alleviates hypoxic endothelial cell death in vitro and protects blood-brain barrier integrity in murine stroke. Cell Death & Disease. 2021;13(1):20.doi:10.1038/s41419-021-04379-z

44.	Duan W L, Wang X J, Ma Y P, Sheng Z M, Dong H, Zhang L Y, et al. Therapeutic strategies targeting the NLRP3‑mediated inflammatory response and pyroptosis in cerebral ischemia/reperfusion injury (Review). Molecular Medicine Reports. 2024;29(3).doi:10.3892/mmr.2024.13170

45.	Zhang M, Liu Z, Zhou W, Shen M, Mao N, Xu H, et al. Ferrostatin-1 attenuates hypoxic-ischemic brain damage in neonatal rats by inhibiting ferroptosis. Translational Pediatrics. 2023;12(11):1944-1970.doi:10.21037/tp-23-189

46.	Hill-Kapturczak N, Thamilselvan V, Liu F, Nick H S, Agarwal A. Mechanism of heme oxygenase-1 gene induction by curcumin in human renal proximal tubule cells. American Journal of Physiology - Renal Physiology. 2001;281(5):F851-859.doi:10.1152/ajprenal.2001.281.5.F851 Deleted and replaced by another article.

47.	Choi A M, Alam J. Heme oxygenase-1: function, regulation, and implication of a novel stress-inducible protein in oxidant-induced lung injury. American Journal of Respiratory Cell and Molecular Biology. 1996;15(1):9-19.doi:10.1165/ajrcmb.15.1.8679227 Deleted.

48.	Wu Z, Uchi H, Morino-Koga S, Shi W, Furue M. Z-ligustilide ameliorated ultraviolet B-induced oxidative stress and inflammatory cytokine production in human keratinocytes through upregulation of Nrf2/HO-1 and suppression of NF-kappaB pathway. Experimental Dermatology. 2015;24(9):703-708.doi:10.1111/exd.12758

49.	Zeng Q, Lian W, Wang G, Qiu M, Lin L, Zeng R. Pterostilbene induces Nrf2/HO-1 and potentially regulates NF-kappaB and JNK-Akt/mTOR signaling in ischemic brain injury in neonatal rats. 3 Biotech. 2020;10(5):192.doi:10.1007/s13205-020-02167-8

50.	Pang Y, Zhang P C, Lu R R, Li H L, Li J C, Fu H X, et al. Andrade-Oliveira Salvianolic Acid B Modulates Caspase-1-Mediated Pyroptosis in Renal Ischemia-Reperfusion Injury via Nrf2 Pathway. Frontiers in Pharmacology. 2020;11:541426.doi:10.3389/fphar.2020.541426

51.	Jia L, Gong Y, Jiang X, Fan X, Ji Z, Ma T, et al. Ginkgolide C inhibits ROS-mediated activation of NLRP3 inflammasome in chondrocytes to ameliorate osteoarthritis. Journal of Ethnopharmacology. 2024;325:117887.doi:10.1016/j.jep.2024.117887

52.	Yan Z, Qi W, Zhan J, Lin Z, Lin J, Xue X, et al. Activating Nrf2 signalling alleviates osteoarthritis development by inhibiting inflammasome activation. Journal of Cellular and Molecular Medicine. 2020;24(22):13046-13057.doi:10.1111/jcmm.15905

53.	Lv C, Cheng T, Zhang B, Sun K, Lu K. Triptolide protects against podocyte injury in diabetic nephropathy by activating the Nrf2/HO-1 pathway and inhibiting the NLRP3 inflammasome pathway. Renal Failure. 2023;45(1):2165103.doi:10.1080/0886022X.2023.2165103

54.	Zhao M W, Yang P, Zhao L L. Chlorpyrifos activates cell pyroptosis and increases susceptibility on oxidative stress-induced toxicity by miR-181/SIRT1/PGC-1alpha/Nrf2 signaling pathway in human neuroblastoma SH-SY5Y cells: Implication for association between chlorpyrifos and Parkinson's disease. Environmental Toxicology. 2019;34(6):699-707.doi:10.1002/tox.22736

55.	Chen Z, Zhong H, Wei J, Lin S, Zong Z, Gong F, et al. Inhibition of Nrf2/HO-1 signaling leads to increased activation of the NLRP3 inflammasome in osteoarthritis. Arthritis Research & Therapy. 2019;21(1):300.doi:10.1186/s13075-019-2085-6

56.	Xu X, Zhang L, Ye X, Hao Q, Zhang T, Cui G, et al. Nrf2/ARE pathway inhibits ROS-induced NLRP3 inflammasome activation in BV2 cells after cerebral ischemia reperfusion. Inflammation Research. 2018;67(1):57-65.doi:10.1007/s00011-017-1095-6

57.	Li M, Tang H, Li Z, Tang W. Emerging Treatment Strategies for Cerebral Ischemia-Reperfusion Injury. Neuroscience. 2022;507:112-124.doi:10.1016/j.neuroscience.2022.10.020

58.	Zhang M, Liu Q, Meng H, Duan H, Liu X, Wu J, et al. Ischemia-reperfusion injury: molecular mechanisms and therapeutic targets. Signal Transduct Target Ther. 2024;9(1):12.doi:10.1038/s41392-023-01688-x

59.	Pell V R, Spiroski A M, Mulvey J, Burger N, Costa A S H, Logan A, et al. Ischemic preconditioning protects against cardiac ischemia reperfusion injury without affecting succinate accumulation or oxidation. J Mol Cell Cardiol. 2018;123:88-91.doi:10.1016/j.yjmcc.2018.08.010

60.	Xu Q, Cheung R T F. Melatonin at repeated doses alleviates hyperglycemia-exacerbated cerebral ischemia-reperfusion injury at 72 h via anti-inflammation and anti-apoptosis. IBRO Neurosci Rep. 2024;16:418-427.doi:10.1016/j.ibneur.2024.03.003

61.	Chae B J, Lee K S, Hwang I, Yu J W. Extracellular Acidification Augments NLRP3-Mediated Inflammasome Signaling in Macrophages. Immune Netw. 2023;23(3):e23.doi:10.4110/in.2023.23.e23

62.	Liu C, Zhang W, Xu G, Zhang D, Zhang C, Qiao S, et al. Deep multilayer brain omics identifies the potential involvement of menopause molecular networks in Gliomas' disease progression. FASEB J. 2022;36(10):e22570.doi:10.1096/fj.202200427RR

63.	Luo L, Song S, Ezenwukwa C C, Jalali S, Sun B, Sun D. Ion channels and transporters in microglial function in physiology and brain diseases. Neurochem Int. 2021;142:104925.doi:10.1016/j.neuint.2020.104925

64.	Kettenmann H, Hanisch U K, Noda M, Verkhratsky A. Physiology of microglia. Physiol Rev. 2011;91(2):461-553.doi:10.1152/physrev.00011.2010 Deleted.

65.	Peretti M, Raciti F M, Carlini V, Verduci I, Sertic S, Barozzi S, et al. Mutual Influence of ROS, pH, and CLIC1 Membrane Protein in the Regulation of G(1)-S Phase Progression in Human Glioblastoma Stem Cells. Mol Cancer Ther. 2018;17(11):2451-2461.doi:10.1158/1535-7163.MCT-17-1223

66.	Zhao R, Zhou X, Zhao Z, Liu W, Lv M, Zhang Z, et al. Farrerol Alleviates Cerebral Ischemia-Reperfusion Injury by Promoting Neuronal Survival and Reducing Neuroinflammation. Molecular Neurobiology. 2024;61(9):7239-7255.doi:10.1007/s12035-024-04031-9

67.	Tang B, Li Y, Xu X, Du G, Wang H. Electroacupuncture Ameliorates Neuronal Injury by NLRP3/ASC/Caspase-1 Mediated Pyroptosis in Cerebral Ischemia-Reperfusion. Molecular Neurobiology. 2024;61(4):2357-2366.doi:10.1007/s12035-023-03712-1

**Reviewer #2 Comments:**

The author has addressed my concerns properly. However, some of the references in the article are dated. It is recommended to add recently published studies on related topics in the field.

Please add the following references:
[1]	Zhao S, Zhuang H, Ji W, Cheng C, Liu Y. Identification of Disulfidptosis-Related Genes in Ischemic Stroke by Combining Single-Cell Sequencing, Machine Learning Algorithms, and In Vitro Experiments. Neuromolecular Medicine. 2024;26(1):39.doi:10.1007/s12017-024-08804-2. PMID: 39278970; PMCID: PMC11402847.

[2]	Qian Y, Yang L, Chen J, Zhou C, Zong N, Geng Y, et al. SRGN amplifies microglia-mediated neuroinflammation and exacerbates ischemic brain injury. Journal of Neuroinflammation‌. 2024;21(1):35.doi:10.1186/s12974-024-03026-6. PMID: 38287411; PMCID: PMC10826034.

[3] Zhuang H, Lei W, Wu Q, Zhao S, Zhao Y, Zhang S, et al. Overexpressed CD73 attenuates GSDMD-mediated astrocyte pyroptosis induced by cerebral ischemia-reperfusion injury through the A2B/NF-kappaB pathway. Experimental Neurology. 2025;386:115152.doi:10.1016/j.expneurol.2025.115152. Epub 2025 Jan 18. PMID: 39832662.

**Responses:**

We would like to thank the reviewers for their positive acknowledgment of our previous revision and for providing additional valuable comments on the manuscript.

We have thoroughly reviewed each reference and made the necessary adjustments. We have replaced or deleted literature prior to 2015 and added reviewer-recommended literatures (labeled in purple). The newly added references have been integrated into the revised manuscript.

We hope these revisions enhance the scientific rigor and clarity of the manuscript.

**Discussion （last paragraph）**

… … Furthermore, a recent study [1] underscores the critical role of the NF-κB-mediated TNF signaling pathway in regulating disulfidptosis-related genes, highlighting its relevance in the pathogenesis of ischemic stroke and potential therapeutic implications. Meanwhile, serglycin was discovered to amplify microglia-mediated neuroinflammation and exacerbate ischemic brain injury by promoting NF-κB signaling and enhancing glucose metabolism in microglia [2]. Additionally, CD73 was found to mitigate GSDMD-mediated astrocyte pyroptosis through the A2B/NF-κB pathway during CIRI [3].

**References (Revised)**

1.	Long Y, Liu S, Wan J, Zhang Y, Li D, Yu S, et al. Brain targeted borneol-baicalin liposome improves blood-brain barrier integrity after cerebral ischemia-reperfusion injury via inhibiting HIF-1alpha/VEGF/eNOS/NO signal pathway. Biomedicine & Pharmacotherapy. 2023;160:114240.doi:10.1016/j.biopha.2023.114240

2.	Premilovac D, Sutherland B A. Acute and long-term changes in blood flow after ischemic stroke: challenges and opportunities. Neural Regeneration Research. 2023;18(4):799-800.doi:10.4103/1673-5374.350699

3.	Wang Z, Li Y, Ye Y, Zhu H, Zhang J, Wang H, et al. NLRP3 inflammasome deficiency attenuates cerebral ischemia-reperfusion injury by inhibiting ferroptosis. Brain Research Bulletin. 2023;193:37-46.doi:10.1016/j.brainresbull.2022.11.016

4.	Liao S, Apaijai N, Chattipakorn N, Chattipakorn S C. The possible roles of necroptosis during cerebral ischemia and ischemia / reperfusion injury. Archives of Biochemistry and Biophysics. 2020;695:108629.doi:10.1016/j.abb.2020.108629

5.	Coimbra-Costa D, Alva N, Duran M, Carbonell T, Rama R. Oxidative stress and apoptosis after acute respiratory hypoxia and reoxygenation in rat brain. Redox Biology. 2017;12:216-225.doi:10.1016/j.redox.2017.02.014

6.	Shi J, Gao W, Shao F. Pyroptosis: Gasdermin-Mediated Programmed Necrotic Cell Death. Trends in Biochemical Sciences. 2017;42(4):245-254.doi:10.1016/j.tibs.2016.10.004

7.	Duan W L, Wang X J, Ma Y P, Sheng Z M, Dong H, Zhang L Y, et al. Therapeutic strategies targeting the NLRP3‑mediated inflammatory response and pyroptosis in cerebral ischemia/reperfusion injury (Review). Molecular Medicine Reports. 2024;29(3).doi:10.3892/mmr.2024.13170

8.	Zhang J Y, Zhou B, Sun R Y, Ai Y L, Cheng K, Li F N, et al. The metabolite alpha-KG induces GSDMC-dependent pyroptosis through death receptor 6-activated caspase-8. Cell Research. 2021;31(9):980-997.doi:10.1038/s41422-021-00506-9

9.	Frank D, Vince J E. Pyroptosis versus necroptosis: similarities, differences, and crosstalk. Cell Death & Differentiation. 2019;26(1):99-114.doi:10.1038/s41418-018-0212-6

10.	Heneka M T, McManus R M, Latz E. Inflammasome signalling in brain function and neurodegenerative disease. Nature Reviews Neuroscience. 2018;19(10):610-621.doi:10.1038/s41583-018-0055-7

11.	Shi S, Zhang C, Liu J. TIMP2 facilitates CIRI through activating NLRP3-mediated pyroptosis. Aging (Albany New York). 2023;15(9):3635-3643.doi:10.18632/aging.204696

12.	Wallach D, Kang T B, Dillon C P, Green D R. Programmed necrosis in inflammation: Toward identification of the effector molecules. Science. 2016;352(6281):aaf2154.doi:10.1126/science.aaf2154

13.	Jiao-Yan Y, Qing-Qing L, Xi L, Mei Z, Ting S, Na H, et al. Oxymatrine improves blood-brain barrier integrity after cerebral ischemia-reperfusion injury by downregulating CAV1 and MMP9 expression. Phytomedicine. 2021;84:153505.doi:10.1016/j.phymed.2021.153505

14.	Uivarosan D, Bungau S, Tit D M, Moisa C, Fratila O, Rus M, et al. Financial Burden of Stroke Reflected in a Pilot Center for the Implementation of Thrombolysis. Medicina (Kaunas). 2020;56(2).doi:10.3390/medicina56020054

15.	Xu M S, Yin L M, Cheng A F, Zhang Y J, Zhang D, Tao M M, et al. Cerebral Ischemia-Reperfusion Is Associated With Upregulation of Cofilin-1 in the Motor Cortex. Frontiers in Cell and Developmental Biology. 2021;9:634347.doi:10.3389/fcell.2021.634347

16.	Salao K, Jiang L, Li H, Tsai V W, Husaini Y, Curmi P M, et al. CLIC1 regulates dendritic cell antigen processing and presentation by modulating phagosome acidification and proteolysis. Biology Open. 2016;5(5):620-630.doi:10.1242/bio.018119

17.	Al Khamici H, Brown L J, Hossain K R, Hudson A L, Sinclair-Burton A A, Ng J P, et al. Members of the chloride intracellular ion channel protein family demonstrate glutaredoxin-like enzymatic activity. PLoS One. 2015;10(1):e115699.doi:10.1371/journal.pone.0115699

18.	Lu D, Le Y, Ding J, Dou X, Mao W, Zhu J. CLIC1 Inhibition Protects Against Cellular Senescence and Endothelial Dysfunction Via the Nrf2/HO-1 Pathway. Cell Biochem Biophys. 2021;79(2):239-252.doi:10.1007/s12013-020-00959-6

19.	Domingo-Fernandez R, Coll R C, Kearney J, Breit S, O'Neill L A J. The intracellular chloride channel proteins CLIC1 and CLIC4 induce IL-1beta transcription and activate the NLRP3 inflammasome. Journal of Biological Chemistry. 2017;292(29):12077-12087.doi:10.1074/jbc.M117.797126

20.	Liu B, Billington C K, Henry A P, Bhaker S K, Kheirallah A K, Swan C, et al. Chloride intracellular channel 1 (CLIC1) contributes to modulation of cyclic AMP-activated whole-cell chloride currents in human bronchial epithelial cells. Physiological Reports. 2018;6(2).doi:10.14814/phy2.13508

21.	Djuric U, Lam K H B, Kao J, Batruch I, Jevtic S, Papaioannou M D, et al. Defining Protein Pattern Differences Among Molecular Subtypes of Diffuse Gliomas Using Mass Spectrometry. Molecular & Cellular Proteomics. 2019;18(10):2029-2043.doi:10.1074/mcp.RA119.001521

22.	Zhang Q, Wang J, Zhang H, Zeng T. Dihydromyricetin inhibits oxidative stress and apoptosis in oxygen and glucose deprivation/reoxygenation‑induced HT22 cells by activating the Nrf2/HO‑1 pathway. Molecular Medicine Reports. 2021;23(6).doi:10.3892/mmr.2021.12036

23.	Wang L, Zhang X, Xiong X, Zhu H, Chen R, Zhang S, et al. Nrf2 Regulates Oxidative Stress and Its Role in Cerebral Ischemic Stroke. Antioxidants (Basel). 2022;11(12).doi:10.3390/antiox11122377

24.	Swanson K V, Deng M, Ting J P. The NLRP3 inflammasome: molecular activation and regulation to therapeutics. Nature Reviews Immunology. 2019;19(8):477-489.doi:10.1038/s41577-019-0165-0

25.	Barbieri F, Verduci I, Carlini V, Zona G, Pagano A, Mazzanti M, et al. Repurposed Biguanide Drugs in Glioblastoma Exert Antiproliferative Effects via the Inhibition of Intracellular Chloride Channel 1 Activity. Frontiers in Oncology. 2019;9:135.doi:10.3389/fonc.2019.00135

26.	Luo L, Song S, Ezenwukwa C C, Jalali S, Sun B, Sun D. Ion channels and transporters in microglial function in physiology and brain diseases. Neurochemistry International. 2021;142:104925.doi:10.1016/j.neuint.2020.104925

27.	Francisco M A, Wanggou S, Fan J J, Dong W, Chen X, Momin A, et al. Chloride intracellular channel 1 cooperates with potassium channel EAG2 to promote medulloblastoma growth. Journal of Experimental Medicine. 2020;217(5).doi:10.1084/jem.20190971

28.	Gururaja Rao S, Patel N J, Singh H. Intracellular Chloride Channels: Novel Biomarkers in Diseases. Frontiers In Physiology. 2020;11:96.doi:10.3389/fphys.2020.00096

29.	Sun X, Cui X. Isorhapontigenin alleviates cerebral ischemia/reperfusion injuries in rats and modulated the PI3K/Akt signaling pathway. Naunyn-Schmiedeberg's Archives of Pharmacology. 2020;393(9):1753-1760.doi:10.1007/s00210-019-01794-0

30.	Zhou S, Gao X, Chen C, Zhang J, Zhang Y, Zhang L, et al. Porcine cardiac blood - Salvia miltiorrhiza root alleviates cerebral ischemia reperfusion injury by inhibiting oxidative stress induced apoptosis through PI3K/AKT/Bcl-2/Bax signaling pathway. Journal of Ethnopharmacology. 2023;316:116698.doi:10.1016/j.jep.2023.116698

31.	Coll R C, Schroder K, Pelegrin P. NLRP3 and pyroptosis blockers for treating inflammatory diseases. Trends in Pharmacological Sciences. 2022;43(8):653-668.doi:10.1016/j.tips.2022.04.003

32.	Carlini V, Verduci I, Cianci F, Cannavale G, Fenoglio C, Galimberti D, et al. CLIC1 Protein Accumulates in Circulating Monocyte Membrane during Neurodegeneration. International Journal of Molecular Sciences. 2020;21(4).doi:10.3390/ijms21041484

33.	Place D E, Kanneganti T D. Recent advances in inflammasome biology. Current Opinion in Immunology. 2018;50:32-38.doi:10.1016/j.coi.2017.10.011

34.	Orekhov A N, Nikiforov N G, Omelchenko A V, Sinyov V V, Sobenin I A, Vinokurov A Y, et al. The Role of Mitochondrial Mutations in Chronification of Inflammation: Hypothesis and Overview of Own Data. Life (Basel). 2022;12(8).doi:10.3390/life12081153

35.	Bellut M, Papp L, Bieber M, Kraft P, Stoll G, Schuhmann M K. NLPR3 inflammasome inhibition alleviates hypoxic endothelial cell death in vitro and protects blood-brain barrier integrity in murine stroke. Cell Death & Disease. 2021;13(1):20.doi:10.1038/s41419-021-04379-z

36.	Chae B J, Lee K S, Hwang I, Yu J W. Extracellular Acidification Augments NLRP3-Mediated Inflammasome Signaling in Macrophages. immune-network. 2023;23(3):e23.doi:10.4110/in.2023.23.e23

37.	Luo J F, Shen X Y, Lio C K, Dai Y, Cheng C S, Liu J X, et al. Activation of Nrf2/HO-1 Pathway by Nardochinoid C Inhibits Inflammation and Oxidative Stress in Lipopolysaccharide-Stimulated Macrophages. Frontiers in Pharmacology. 2018;9:911.doi:10.3389/fphar.2018.00911

38.	Wu Z, Uchi H, Morino-Koga S, Shi W, Furue M. Z-ligustilide ameliorated ultraviolet B-induced oxidative stress and inflammatory cytokine production in human keratinocytes through upregulation of Nrf2/HO-1 and suppression of NF-kappaB pathway. Experimental Dermatology. 2015;24(9):703-708.doi:10.1111/exd.12758

39.	Zeng Q, Lian W, Wang G, Qiu M, Lin L, Zeng R. Pterostilbene induces Nrf2/HO-1 and potentially regulates NF-kappaB and JNK-Akt/mTOR signaling in ischemic brain injury in neonatal rats. 3 Biotech. 2020;10(5):192.doi:10.1007/s13205-020-02167-8

40.	Pang Y, Zhang P C, Lu R R, Li H L, Li J C, Fu H X, et al. Andrade-Oliveira Salvianolic Acid B Modulates Caspase-1-Mediated Pyroptosis in Renal Ischemia-Reperfusion Injury via Nrf2 Pathway. Frontiers in Pharmacology. 2020;11:541426.doi:10.3389/fphar.2020.541426

41.	Jia L, Gong Y, Jiang X, Fan X, Ji Z, Ma T, et al. Ginkgolide C inhibits ROS-mediated activation of NLRP3 inflammasome in chondrocytes to ameliorate osteoarthritis. Journal of Ethnopharmacology. 2024;325:117887.doi:10.1016/j.jep.2024.117887

42.	Yan Z, Qi W, Zhan J, Lin Z, Lin J, Xue X, et al. Activating Nrf2 signalling alleviates osteoarthritis development by inhibiting inflammasome activation. Journal of Cellular and Molecular Medicine. 2020;24(22):13046-13057.doi:10.1111/jcmm.15905

43.	Lv C, Cheng T, Zhang B, Sun K, Lu K. Triptolide protects against podocyte injury in diabetic nephropathy by activating the Nrf2/HO-1 pathway and inhibiting the NLRP3 inflammasome pathway. Renal Failure. 2023;45(1):2165103.doi:10.1080/0886022X.2023.2165103

44.	Zhao M W, Yang P, Zhao L L. Chlorpyrifos activates cell pyroptosis and increases susceptibility on oxidative stress-induced toxicity by miR-181/SIRT1/PGC-1alpha/Nrf2 signaling pathway in human neuroblastoma SH-SY5Y cells: Implication for association between chlorpyrifos and Parkinson's disease. Environmental Toxicology. 2019;34(6):699-707.doi:10.1002/tox.22736

45.	Chen Z, Zhong H, Wei J, Lin S, Zong Z, Gong F, et al. Inhibition of Nrf2/HO-1 signaling leads to increased activation of the NLRP3 inflammasome in osteoarthritis. Arthritis Research & Therapy. 2019;21(1):300.doi:10.1186/s13075-019-2085-6

46.	Xu X, Zhang L, Ye X, Hao Q, Zhang T, Cui G, et al. Nrf2/ARE pathway inhibits ROS-induced NLRP3 inflammasome activation in BV2 cells after cerebral ischemia reperfusion. Inflammation Research. 2018;67(1):57-65.doi:10.1007/s00011-017-1095-6

47.	Li L, Dickinson M S, Coers J, Miao E A. Pyroptosis in defense against intracellular bacteria. Semin Immunol. 2023;69:101805.doi:10.1016/j.smim.2023.101805

48.	Li M, Tang H, Li Z, Tang W. Emerging Treatment Strategies for Cerebral Ischemia-Reperfusion Injury. Neuroscience. 2022;507:112-124.doi:10.1016/j.neuroscience.2022.10.020

49.	Zhang M, Liu Q, Meng H, Duan H, Liu X, Wu J, et al. Ischemia-reperfusion injury: molecular mechanisms and therapeutic targets. Signal Transduction and Targeted Therapy. 2024;9(1):12.doi:10.1038/s41392-023-01688-x

50.	Pell V R, Spiroski A M, Mulvey J, Burger N, Costa A S H, Logan A, et al. Ischemic preconditioning protects against cardiac ischemia reperfusion injury without affecting succinate accumulation or oxidation. J Mol Cell Cardiol. 2018;123:88-91.doi:10.1016/j.yjmcc.2018.08.010

51.	Xu Q, Cheung R T F. Melatonin at repeated doses alleviates hyperglycemia-exacerbated cerebral ischemia-reperfusion injury at 72 h via anti-inflammation and anti-apoptosis. Ibro Neuroscience Reports. 2024;16:418-427.doi:10.1016/j.ibneur.2024.03.003

52.	Peretti M, Raciti F M, Carlini V, Verduci I, Sertic S, Barozzi S, et al. Mutual Influence of ROS, pH, and CLIC1 Membrane Protein in the Regulation of G(1)-S Phase Progression in Human Glioblastoma Stem Cells. Molecular Cancer Therapeutics. 2018;17(11):2451-2461.doi:10.1158/1535-7163.MCT-17-1223

53.	Liu C, Zhang W, Xu G, Zhang D, Zhang C, Qiao S, et al. Deep multilayer brain omics identifies the potential involvement of menopause molecular networks in Gliomas' disease progression. FASEB Journal. 2022;36(10):e22570.doi:10.1096/fj.202200427RR

54.	Zhao R, Zhou X, Zhao Z, Liu W, Lv M, Zhang Z, et al. Farrerol Alleviates Cerebral Ischemia-Reperfusion Injury by Promoting Neuronal Survival and Reducing Neuroinflammation. Molecular Neurobiology. 2024;61(9):7239-7255.doi:10.1007/s12035-024-04031-9

55.	Zhao S, Zhuang H, Ji W, Cheng C, Liu Y. Identification of Disulfidptosis-Related Genes in Ischemic Stroke by Combining Single-Cell Sequencing, Machine Learning Algorithms, and In Vitro Experiments. Neuromolecular Medicine. 2024;26(1):39.doi:10.1007/s12017-024-08804-2

56.	Qian Y, Yang L, Chen J, Zhou C, Zong N, Geng Y, et al. SRGN amplifies microglia-mediated neuroinflammation and exacerbates ischemic brain injury. Journal of Neuroinflammation‌. 2024;21(1):35.doi:10.1186/s12974-024-03026-6

57.	Zhuang H, Lei W, Wu Q, Zhao S, Zhao Y, Zhang S, et al. Overexpressed CD73 attenuates GSDMD-mediated astrocyte pyroptosis induced by cerebral ischemia-reperfusion injury through the A2B/NF-kappaB pathway. Experimental Neurology. 2025;386:115152.doi:10.1016/j.expneurol.2025.115152
