# Supplementary material for: CLIC1 down-regulates Nrf2/HO-1 signalling pathway promoting the apoptosis and pyroptosis in OGD/R-treated HT22 cells
Source: PLoS One. 2025 Sep 18;20(9):e0332698. doi: 10.1371/journal.pone.0332698 (PMC12445528; doi:10.1371/journal.pone.0332698)
Supplement: Attachment — Submitted filename: Response to Reviewers_2025.7.docx [file pone.0332698.s010.docx]

**Dear Editor and Reviewer,**

We sincerely appreciate the time and effort you have dedicated to reviewing our manuscript. We are grateful for the rigorous, professional, and constructive feedback provided throughout the review process.

Our manuscript has undergone two rounds of revision—a major revision (PONE-D-24-49743_R1) and a minor revision (PONE-D-24-49743_R2)—prior to this submission. However, we noticed that the latest reviewer’s comments appear to be based on the original version of the manuscript (PONE-S-24-63293) rather than the most recently revised version.

Given that the previous reviewers and editor have already provided valuable suggestions to enhance the language, literature citations, readability, logical flow, and scientific rigor of the paper, we have made further refinements based on the latest revised version (PONE-D-24-49743_R2) rather than reverting to the original submission.

To facilitate your review, we have:

- Included a point-by-point response to all comments in this letter.
- Highlighted all new revisions in yellow in the revised manuscript.

We deeply value your expertise and the thoughtful critique, which has significantly strengthened our work. Should you have any additional questions or concerns, please do not hesitate to contact us.

Thank you once again for your time and consideration.

Best regards,

Chuang Sun

**Journal requirements:**

Comment #1

We note that one or more reviewers has recommended that you cite specific previously published works in an earlier round of revision. As always, we recommend that you please review and evaluate the requested works to determine whether they are relevant and should be cited. It is not a requirement to cite these works and you may remove them before the manuscript proceeds to publication. We appreciate your attention to this request.

Thank you for your note regarding the suggested citations from the reviewers. We have carefully evaluated the recommended references in relation to our study’s scope and focus.

After thorough consideration, we determined that the suggested works are not directly applicable to our research objectives.

Below is the excluded documents and related content:

**Discussion** **Penultimate paragraph** (PONE-D-24-49743_R2)

… Furthermore, a recent study [55] underscores the critical role of the NF-κB-mediated TNF signaling pathway in regulating disulfidptosis-related genes, highlighting its relevance in the pathogenesis of ischemic stroke and potential therapeutic implications. Meanwhile, serglycin was discovered to amplify microglia-mediated neuroinflammation and exacerbate ischemic brain injury by promoting NF-κB signaling and enhancing glucose metabolism in microglia[56]. Additionally, CD73 was found to mitigate GSDMD-mediated astrocyte pyroptosis through the A2B/NF-κB pathway during CIRI[57].

[55] Zhao S, Zhuang H, Ji W, Cheng C, Liu Y. Identification of Disulfidptosis-Related Genes in Ischemic Stroke by Combining Single-Cell Sequencing, Machine Learning Algorithms, and In Vitro Experiments. Neuromolecular Medicine. 2024;26(1):39.doi:10.1007/s12017-024-08804-2

[56] Qian Y, Yang L, Chen J, Zhou C, Zong N, Geng Y, et al. SRGN amplifies microglia-mediated neuroinflammation and exacerbates ischemic brain injury. Journal of Neuroinflammation. 2024;21(1):35.doi:10.1186/s12974-024-03026-6

[57] Zhuang H, Lei W, Wu Q, Zhao S, Zhao Y, Zhang S, et al. Overexpressed CD73 attenuates GSDMD-mediated astrocyte pyroptosis induced by cerebral ischemia-reperfusion injury through the A2B/NF-kappaB pathway. Experimental Neurology. 2025;386:115152.doi:10.1016/j.expneurol.2025.115152

**Response to reviewer:**

Comment #1

How do the authors reconcile the fact that manipulating the same molecule CLIC1 may lead to two distinct cell death processes – apoptosis and pyroptosis that can not occur at the same time. Is there a preferential pathway to one or the other, or a state of the cell or a time window?

We sincerely appreciate the reviewer's thoughtful question regarding the distinct cell death pathways (apoptosis and pyroptosis) mediated by CLIC1 manipulation. This is indeed a crucial aspect of our findings that warrants careful discussion.

**1. Temporal Dynamics:**

Our findings suggest a mechanistic that OGD/R triggers upregulation of CLIC1, leading to suppression of the Nrf2/HO-1 antioxidant signaling pathway. These processes results in increased ROS accumulation and disturbance of redox balance, promoting apoptosis. Additionally, elevated CLIC1 activates the NLRP3 inflammasome and caspase-1, inducing pyroptosis via upregulation of pyroptosis markers. **Although the downstream cell death modalities differ, CLIC1 primarily exerts its effect by creating a pro-oxidative environment that is permissive for both pathways.**

**While apoptosis is often described as a non-inflammatory form of cell death, it has the capacity to trigger mild inflammatory responses[1].** Apoptosis aids in clearing infected cells by phagocytes through efferocytosis, whereas pyroptosis, driven by the inflammasome, facilitates the removal of intracellular pathogens and the activation of immune responses[2]. As shown in Figures 6 (revised) and 7, we found that activation of NLRP3 in OGD/R-treated HT22 cells result in increased apoptosis rates (Fig. 6a, b) and higher mean fluorescence intensity (MFI) of ROS (Fig. 6a, c). Additionally, NLRP3 activation exacerbates oxidative stress by increasing oxidative stress–related indices of SOD, CAT, GSH-PX, CCK-8, LDH, Bal-2 and Bax (Fig. 7). **These data imply that NLRP3-driven pyroptosis can in turn amplify oxidative stress and apoptotic processes, indicating these events may occur in a sequential or partially overlapping manner rather than as mutually exclusive forms of cell death.** Since this finding is not a major finding, we did not explain it in the Figure Legend, but rather inside the **Revised Results.**

**Figures 6 (revised)**

**Fig 6. CLIC1 exacerbates apoptosis and reactive oxygen species (ROS) accumulation in oxygen and glucose deprivation/reoxygenation (OGD/R)-treated HT22 cells by inhibiting the Nrf2/HO-1 signalling pathway.** Flow cytometry analysis shows that inhibition of the Nrf2/HO-1 signalling pathway increased both the apoptosis rate (a, b) and ROS mean fluorescence intensity (MFI) value (a, c) in OGD/R-treated HT22 cells. These levels were higher in the OGD/R+CLIC1 silencing+Nrf2/HO-1 inhibition group compared to the OGD/R+CLIC1 silencing group.

**Fig 7. CLIC1 accelerates oxidative stress in oxygen and glucose deprivation/reoxygenation (OGD/R)-treated HT22 cells by inhibiting the Nrf2/HO-1 signalling pathway.** (a-c) Biochemical analysis of oxidative stress markers showed reduced activity of SOD (a), CAT (b), and GSH-Px (c) following inhibition of the Nrf2/HO-1 signalling pathway. These reductions were more pronounced in the OGD/R+CLIC1 silencing+Nrf2/HO-1 inhibition group compared to the OGD/R+CLIC1 silencing group. (d) CCK-8 OD values were decreased after Nrf2/HO-1 inhibition, with lower values observed in the OGD/R+CLIC1 silencing+Nrf2/HO-1 inhibition group compared to the OGD/R+CLIC1 silencing group. (e) The LDH levels were increased after Nrf2/HO-1 inhibition and were significantly higher in the OGD/R+CLIC1 silencing+Nrf2/HO-1 inhibition group compared to the OGD/R+CLIC1 silencing group. (f-h) Real-time PCR (f) and WB (g, h) analyses showed that Bcl-2 expression was downregulated, while Bax expression was upregulated following Nrf2/HO-1 inhibition. These effects were more pronounced in the OGD/R+CLIC1 silencing+Nrf2/HO-1 inhibition group compared to the OGD/R+CLIC1 silencing group. Data were presented as mean ± SEM, with n = 3 per group. *P <0.05 versus control group; ^P <0.05 versus OGD/R group; +P <0.05 vs OGD/R+CLIC1 silencing group.

**Revised Results (paragraph 5)**

**CLIC1 inhibits the Nrf2/HO-1 signalling pathway to accelerate pyroptosis**

As explained above, the increased CLIC1 led to decreased Nrf2/HO-1 expression. We also found that the expression levels of inflammation- and pyroptosis-related indicators NLRP3, caspase-1, caspase-3, GSDMD, IL-1β, and IL-18 were all increased after inhibiting the Nrf2/HO-1 pathway in HT22 cells treated with OGD/R, and their levels were higher in the OGD/R+CLIC1 silencing+ Nrf2/HO-1 inhibition group than in the OGD/R+CLIC1 silencing group (Fig 4). Thus, NLRP3-mediated pyroptosis could be accelerated by inhibiting the Nrf2/HO-1 signalling pathway. Additionally, activation of NLRP3 in OGD/R-treated HT22 cells led to increased apoptosis rates (Fig. 6a, b) and higher mean fluorescence intensity (MFI) of ROS (Fig. 6a, c). NLRP3 activation also exacerbates oxidative stress by regulating oxidative stress–related indices of SOD, CAT, GSH-PX, CCK-8, LDH, Bal-2 and Bax (Fig. 7). These data imply that NLRP3-driven pyroptosis can in turn amplify oxidative stress and apoptotic processes, indicating these events may occur in a sequential or partially overlapping manner rather than as mutually exclusive forms of cell death.

**2. Cellular Heterogeneity:**

Our experiments measure population-wide signals, and the apparent co-activation of both death pathways may reflect different subpopulations of cells—some undergoing apoptosis, others pyroptosis. The correlation of CLIC1 with markers of both processes indicates its central role in creating the stress environment needed for either pathway, not necessarily the simultaneous occurrence in all cells.

**3. Evidence for Transition Between Pyroptosis and Apoptosis:**

**The dynamic interplay between apoptosis and pyroptosis in response to oxidative injury is receiving growing recognition across various cellular injury models. Recent researches have demonstrated the possibility of transition and crosstalk between pyroptosis and apoptosis mechanisms.** For example, Yuan et al. [3]found that the saponin monomer 13 of dwarf lilyturf tuber (DT-13) inhibits methylglyoxal-induced pyroptosis and facilitates a shift toward apoptosis by specifically modulating Caspase3/gasdermin E pathway in human umbilical vein endothelial cells. This underscores the plasticity between these cell death forms. Cao et al.[4] demonstrates that under diabetic and high glucose conditions, unfolded protein response pathways and the NLRP3 inflammasome are activated in podocytes, promoting both apoptosis and pyroptosis, and thioredoxin-interacting protein serves as a key link that mediates the mutual transformation between these two forms of cell death.

**In summary, apoptosis and pyroptosis play distinct yet overlapping roles in both eliminating damaged cells and activating immune responses to CIRI.** However, their relative contributions in CIRI depend on variables such as the specific experimental model used, the severity and duration of ischemia, and the extent of reperfusion injury. **Considering this complexity, therapeutic strategies that integrate both anti-inflammatory and antioxidant or transformation of cell death forms hold great potential for managing CIRI.**

We have further revised and summarized these in the **Revised Discussion.**

**Revised Discussion (paragraph 5)**

While apoptosis is often described as a non-inflammatory form of cell death, it has the capacity to trigger mild inflammatory responses[1]. Apoptosis aids in clearing infected cells by phagocytes through efferocytosis, whereas pyroptosis, driven by the inflammasome, facilitates the removal of intracellular pathogens and the activation of immune responses[2]. We found that NLRP3-driven pyroptosis can in turn amplify oxidative stress and apoptotic processes, by regulating oxidative stress–related indices, MFI of ROS and apoptosis rates. Together, these cell death mechanisms play distinct yet overlapping roles in both eliminating damaged cells and activating immune responses to CIRI. Notably, our experiments measure population-wide signals, and the apparent co-activation of both death pathways may reflect different subpopulations of cells—some undergoing apoptosis, others pyroptosis. The correlation of CLIC1 with markers of both processes further indicates its central role in creating the stress environment needed for either pathway, not necessarily the simultaneous occurrence in all cells. Additionally, recent research has demonstrated the possibility of transition and crosstalk between pyroptosis and apoptosis mechanisms. For example, Yuan et al. [3]found that the saponin monomer 13 of dwarf lilyturf tuber (DT-13) inhibits methylglyoxal-induced pyroptosis and facilitates a shift toward apoptosis by specifically modulating Caspase3/gasdermin E pathway in human umbilical vein endothelial cells. This underscores the plasticity between these cell death forms. Cao et al.[4] demonstrates that under diabetic and high glucose conditions, unfolded protein response pathways and the NLRP3 inflammasome are activated in podocytes, promoting both apoptosis and pyroptosis, and thioredoxin-interacting protein serves as a key link that mediates the mutual transformation between these two forms of cell death. However, apoptosis and pyroptosis relative contributions in CIRI depend on variables such as the specific experimental model used, the severity and duration of ischemia, and the extent of reperfusion injury. Considering this complexity, therapeutic strategies that integrate both anti-inflammatory and antioxidant or transformation of cell death forms hold great potential for managing CIRI.

Comment #2

What is the rationale for the CLIC1 multiple roles in inflammation and oxidative stress in ischemic damage. Using the term „cellular adaptor“ (Discussion- line 5) will not do.

**1.** Thank you for raising this important point. We provide the following explanation, with additional clarifications in the **Revised Discussion** of our manuscript.

**Chloride intracellular channel 1 (CLIC1) is a multifunctional protein expressed in various organs, where it participates in enzymatic activity, redox regulation and inflammatory processes [5, 6]** . CLIC1 is an oxidative stress sensor in vascular endothelium cells [7]. In response to increased cytoplasmic oxidation or changes in pH, cytoplasmic soluble CLIC proteins integrate into cellular membranes, functioning as anion channels to counteract cationic fluctuations [8]. CLIC1 was found to modulate cyclic adenosine monophosphate (cAMP)-induced chloride currents in bronchial epithelial cells, maintaining chloride homeostasis [9]. **Our study for the first time revealed that the level of CLICI is upregulated in the OGD/R-treated HT22 cell model of CIRI, suggesting its involvement in the pathological processes of ischemia-reperfusion injury. The upregulation of CLIC1 in OGD/R-treated HT22 cells appears to be closely associated with oxidative stress and the inflammatory response—two key drivers of neuronal damage in CIRI.**

**(1) Oxidative Stress:** During reperfusion, CLIC1 may be upregulated as part of the cellular defence mechanism to counteract oxidative damage and regulate intracellular chloride levels. However, the overexpression of CLIC1 disrupts redox homeostasis by promoting ROS generation and altering mitochondrial function. By interacting with specific proteins, CLIC1 was reported to promote mitochondrial fragmentation, increases membrane potential and ROS production, and induces a metabolic shift toward glycolysis in endothelial cells[10]. Whereas, inhibition of CLIC1 reduced oxidative stress-induced damage by lowering ROS levels, decreasing intercellular adhesion molecule 1 (ICAM1) and vascular cell adhesion molecule 1 (VCAM1), and enhancing the activity of antioxidant enzymes, including SOD [7]. Furthermore, we observed that CLIC1 regulated apoptotic markers, such as the pro-apoptotic Bax and anti-apoptotic Bcl-2, highlight its role in apoptosis process in OGD/R-treated HT22 cells. Oxidative stress-induced apoptosis in CIRI can be alleviated by modulating the Bcl-2/Bax signalling pathway, which involves the downregulation of Bax and upregulation of Bcl-2 [11, 12]. These findings suggest that CLIC1 may contribute to CIRI by fostering a redox imbalance and promoting apoptosis.

**(2) Inflammation:** CLIC1 has been shown to translocate to the plasma membrane upon cellular activation, where it modulates ion homeostasis and cell signaling, including the production and release of pro-inflammatory cytokines. A previous study[8]demonstrated that CLIC1 and CLIC4 can regulate the NLRP3 inflammasome, with ‌Lipopolysaccharide stimulation prompting their translocation from the cytoplasm to the nucleus and cell membrane in macrophages. While knockdown of CLIC1 and CLIC4 impairs IL-1β transcription, apoptosis-associated speck-like protein containing a CARD （ASC） speck formation, and secretion of mature IL-1β, indicating their essential role in inflammasome activation, possibly via Cl⁻ efflux. Furthermore, Carlini et al.[13] revealed that CLIC1 levels were significantly elevated in peripheral blood mononuclear cells during chronic central nervous system inflammation, suggesting its potential involvement in inflammatory processes and act as a potential marker of neurodegenerative processes.

**Revised Discussion**

**Paragraph 2**

CLIC1 is a metamorphic protein that exists in both soluble cytoplasmic and membrane-associated forms, with the latter functioning as a chloride-selective ion channel[14]. It plays a key role in physiological processes such as stabilizing membrane potential, regulating pH, cell proliferation, fluid secretion, and maintaining cell volume[7, 15]. Previous research has reported elevated CLIC1 levels in various neurological disorders and central nervous system cancers, such as Alzheimer’s disease (AD), medulloblastoma, and gliomas [16-18]. During reperfusion, CLIC1 may be upregulated as part of the cellular defence mechanism to counteract oxidative damage and regulate intracellular chloride levels. However, the overexpression of CLIC1 disrupts redox homeostasis by promoting ROS generation and altering mitochondrial function. By interacting with specific proteins, CLIC1 was reported to promote mitochondrial fragmentation, increases membrane potential and ROS production, and induces a metabolic shift toward glycolysis in endothelial cells[10]. Whereas, inhibition of CLIC1 reduced oxidative stress-induced damage by lowering ROS levels, decreasing intercellular adhesion molecule 1 (ICAM1) and vascular cell adhesion molecule 1 (VCAM1), and enhancing the activity of antioxidant enzymes, including SOD [7]. Furthermore, we observed that CLIC1 regulated apoptotic markers, such as the pro-apoptotic Bax and anti-apoptotic Bcl-2, highlight its role in apoptosis process in OGD/R-treated HT22 cells. Oxidative stress-induced apoptosis in CIRI can be alleviated by modulating the Bcl-2/Bax signalling pathway, which involves the downregulation of Bax and upregulation of Bcl-2 [11, 12]. These findings suggest that CLIC1 may contribute to CIRI by fostering a redox imbalance and promoting apoptosis.

**Paragraph 3**

Concurrently, we found that CLIC1 upregulation promoted NLRP3-mediated pyroptosis in OGD/R-treated HT22 cells. CLIC1 has been shown to translocate to the plasma membrane upon cellular activation, where it modulates ion homeostasis and cell signaling, including the production and release of pro-inflammatory cytokines. A previous study[8]demonstrated that CLIC1 and CLIC4 can regulate the NLRP3 inflammasome, with ‌Lipopolysaccharide stimulation prompting their translocation from the cytoplasm to the nucleus and cell membrane in macrophages. While knockdown of CLIC1 and CLIC4 impairs IL-1β transcription, apoptosis-associated speck-like protein containing a CARD （ASC） speck formation, and secretion of mature IL-1β, indicating their essential role in inflammasome activation, possibly via Cl⁻ efflux. Furthermore, Carlini et al.[13] revealed that CLIC1 levels were significantly elevated in peripheral blood mononuclear cells during chronic central nervous system inflammation, suggesting its potential involvement in inflammatory processes and act as a potential marker of neurodegenerative processes. ....

**2.** We appreciate the reviewer’s concern that the term **"cellular adaptor"** alone may not fully capture the mechanistic basis of CLIC1’s function. We have revised this sentence in Discussion section to provide a more mechanistic explanation of CLIC1’s roles in inflammation and oxidative stress, emphasizing its biophysical properties, ability to translocate, and function as a signaling mediator rather than simply a cellular adaptor.

**Original Discussion (Paragraph 1, line 5)**

Our study delved into the role of chloride intracellular channel 1 (CLIC1) in modulating cellular responses to oxidative stress and inflammation in HT22 cells under OGD/R conditions. Firstly, we observed that CLIC1 expression was significantly elevated in OGD/R-treated HT22 cells. This increase underscores the potential role of CLIC1 as a stress-responsive protein, possibly acting as a cellular adaptor in conditions of metabolic stress. Notably, following targeted silencing of CLIC1, its expression was effectively downregulated, demonstrating the modulation achievable through genetic interventions….

**Revised Discussion (Paragraph 2)**

CLIC1 is a metamorphic protein that exists in both soluble cytoplasmic and membrane-associated forms, with the latter functioning as a chloride-selective ion channel[14]. It plays a key role in physiological processes such as stabilizing membrane potential, regulating pH, cell proliferation, fluid secretion, and maintaining cell volume[7, 15]. …

Comment #3

It is never disclosed what is the basis of the hypothesis that CLIC1 would suppress Nrf2/HO-1. In fact, it is not clearly disclosed what is the initial downstream pathway for the many CLIC1 effects. Only in Fig 1 “ARE” is indicated without further mentioning or even explaining the abbreviation.

Thank you for highlighting the need to better emphasize the novel contribution of our study. We have undertaken revisions to strengthen the emphasis on this point throughout the manuscript as follows:

**1. Basis of the hypothesis and initial downstream pathways for CLIC1 effects:**

CLIC1 functions as an oxidative stress sensor and has been reported to regulate cellular senescence and function via the Nrf2/HO-1 pathway [7]. However, the precise initial downstream mechanisms remain unclear. Currently，evidence suggests that CLIC1 inhibition increases intracellular Ca²⁺ via L-type calcium channel  (LTCC) [19], while conformational changes in CLIC1 can modulate Nrf2 signaling by altering Ca²⁺ homeostasis[20]. Additionally, CLIC1 overexpression inhibits Nrf2 nuclear translocation and contributes to hydrogen peroxide-induced mitochondrial dysfunction and fission[7]. Nrf2 functions as a key antioxidant transcription factor by promoting the expression of detoxification and antioxidant enzymes, including HO-1 [7, 21]. Thus, these events disrupt membrane potential and calcium balance, promote ROS generation, impair mitochondrial function, and ultimately reduce Nrf2 activation and HO-1 expression.

**Revised Discussion (paragraph 4)**

CLIC1 functions as an oxidative stress sensor and has been reported to regulate cellular senescence and function via the Nrf2/HO-1 pathway [7]. However, the precise initial downstream mechanisms remain unclear. Evidence suggests that CLIC1 inhibition increases intracellular Ca²⁺ via LTCC) [19], while conformational changes in CLIC1 can modulate Nrf2 signaling by altering Ca²⁺ homeostasis[20]. Additionally, CLIC1 overexpression inhibits Nrf2 nuclear translocation and contributes to hydrogen peroxide-induced mitochondrial dysfunction and fission[7]. As Nrf2 functions as a key antioxidant transcription factor by promoting the expression of detoxification and antioxidant enzymes, including HO-1 [7, 21], these events ultimately reduce Nrf2 activation and HO-1 expression….

**2. Explanation and definition of “ARE”:**
We regret for not defining “ARE” (antioxidant response element). We have revised both the manuscript and Figure 1 legend to clearly define “ARE” as follows:

**Revised introduction (end of paragraph 3)**

… Nrf2 bind to antioxidant response element (ARE), a specific DNA sequence located in the promoter regions of genes that encode antioxidant and cytoprotective proteins, in response to oxidative or electrophilic stress, activating the expression of these genes and enhancing the cell’s defense mechanisms against damage[22]. Nrf2/HO-1 pathway is a key protector against oxidative stress-mediated apoptosis and related to inflammasome regulation and pyroptosis [23-25].

**Revised Figure 1 and legend**

**Fig 1. Schematic illustration of the hypothesis of CLIC1 mediates neuronal injury following OGD/R in HT22 cells.**

Mouse hippocampal neurons (modelled using HT22 cells) are subjected to OGD/R to mimic CIRI. OGD/R triggers upregulation of CLIC1, which leads to suppression of the Nrf2/HO-1 antioxidant signaling pathway. This results in increased ROS accumulation and disturbance of redox balance, promoting apoptosis by increased Bax and decreased Bcl-2 expression. Additionally, elevated CLIC1 activates the NLRP3 inflammasome and caspase-1, inducing pyroptosis via upregulation of pyroptosis markers, such as caspase-3, GSDMD, IL-1β, and IL-18. Collectively, these processes contribute to neuronal cell damage after CIRI. OGD/R, oxygen-glucose deprivation/reoxygenation; CIRI, cerebral ischemia-reperfusion injury; CLIC1, chloride intracellular channel 1; Nrf2, nuclear factor erythroid 2–related factor 2; ARE, antioxidant response element; HO-1, heme oxygenase 1; ROS, reactive oxygen species; NLRP3, NOD-like receptor family pyrin domain containing 3; GSDMD, gasdermin D.

Comment #4

How do the authors reconcile their proposal of therapy by supressing CLIC1 and apoptosis/pyroptosis with their stating that CLIC1 has a cellular defensive mechanism (p. 16, 2pgph line 2-3) or that it stabilizes cation fluctuations during stress conditions and cytoplasmic oxidation (p17 last line, p18 lines 1-2) as well as with the sentence „Apoptosis aids in clearing infected cells, whereas pyroptosis facilitates the removal of intracellular pathogens and the activation of immune responses.

**Original manuscript**

p. 16, 2pgph line 2-3: Firstly, ischemia and subsequent reperfusion result in a burst of reactive oxygen species (ROS) during reperfusion, leading to oxidative stress. CLIC1 is upregulated as part of a cellular defensive mechanism to manage increased intracellular chloride levels and oxidative stress[26].

p17 last line, p18 lines 1-2 Additionally, ion homeostasis disruption upon reperfusion can lead to cellular swelling and damage. CLIC1 regulates chloride ion transport, which is essential for maintaining osmotic balance and cell volume during stress conditions[27]. During cytoplasmic oxidation or pH changes, soluble CLIC proteins translocate to the cell membrane, functioning as anion channels to stabilize cation fluctuations[8].

[28]	Djuric U, Lam KHB, Kao J, Batruch I, Jevtic S, Papaioannou MD, et al. Defining Protein Pattern Differences Among Molecular Subtypes of Diffuse Gliomas Using Mass Spectrometry. Mol Cell Proteomics. 2019;18(10):2029-43.doi:10.1074/mcp.RA119.001521

[29]	Ulmasov B, Bruno J, Woost PG, Edwards JC. Tissue and subcellular distribution of CLIC1. BMC Cell Biol. 2007;8:8.doi:10.1186/1471-2121-8-8

[8] Zhang JY, Zhou B, Sun RY, Ai YL, Cheng K, Li FN, et al. The metabolite alpha-KG induces GSDMC-dependent pyroptosis through death receptor 6-activated caspase-8. Cell Res. 2021;31(9):980-97.doi:10.1038/s41422-021-00506-9

We appreciate the reviewer’s thoughtful question. Our proposal to target CLIC1 suppression as a therapeutic strategy, despite its role in cellular defense and ion homeostasis. This can be reconciled by considering the following CLIC1 activities:

**1. Context-dependent functions:**

CLIC1’s defensive functions (e.g., managing oxidative stress, stabilizing cation fluctuations) are likely beneficial in early or moderate stress, where it aids in maintaining homeostasis. However, in severe pathological conditions (e.g., sustained ischemia-reperfusion injury or chronic inflammation), CLIC1 may become overactive or dysregulated, exacerbating apoptosis/pyroptosis and tissue damage. Here, suppression could limit excessive cell death or immune hyper-activation.

**2. Therapeutic Balance:**

Our therapeutic proposal suggestion is to temporally or spatially control CLIC1 inhibition—targeting its harmful effects (e.g., pro-death signaling) while preserving its protective roles. **The reconciliation lies in the idea that CLIC1’s role shifts from protective to harmful depending on the disease stage or severity, and therapeutic targeting would aim to inhibit its detrimental effects without entirely abolishing its physiological functions.** This approach is common in therapies targeting proteins.

Although there are no specific studies directly focusing on CLIC1 inhibitors for CIRI, previous studies [15, 28] have suggested CLIC1 as a potential therapeutic target for central nervous system diseases, including brain tumors and neurodegenerative diseases. For example, inhibition of CLIC1 channels using specific blockers may effectively suppress microglial proliferation and mitigated the neurotoxicity induced by Aβ-treated microglial cells [15]. Meanwhile, CLIC1 was positively correlated with infiltration of immune cells and significantly associated with worse overall survival in glioma patients[29]. CLIC1 is highly expressed in the cancer stem cells (CSCs) of glioblastoma, and modulates the tumor microenvironment, promoting cancer cell proliferation and invasion. Whereas, inhibition of CLIC1 significantly induces G1 phase cell cycle arrest in glioblastoma CSCs, and the functional impairment of CLIC1 on the membrane causes glioblastoma CSCs to shift from an allostatic tumorigenic state to a homeostatic steady state [30]. All these findings suggest that CLIC1 is a critical therapeutic target for central nervous system disorders and holds promising potential for treating CIRI.

We have revised in the **Discussion** about this comment.

**Revised Discussion paragraph 6**

Currently, the primary long-term objective of CIRI therapy aims to restore blood flow, reduce secondary damage, and minimize stroke-related neuronal cell death, disability, and mortality [31]. The treatment strategies for CIRI mainly include pre-ischemic preconditioning, post-ischemic preconditioning, and pharmacological preconditioning. Various pharmacological and interventional therapies have been developed to mitigate CIRI. However, the complexity of CIRI and the intricate interplay among various signalling pathways present significant challenges, leading to a lack of consensus in existing research [32, 33]. Neuroprotective measures such as antioxidants and anti-inflammatory agents have shown promise in preclinical CIRI models [34]. Although there are no specific studies directly focusing on CLIC1 inhibitors for CIRI, previous studies [15, 28] have suggested CLIC1 as a potential therapeutic target for central nervous system diseases, including brain tumours and neurodegenerative diseases. For example, inhibition of CLIC1 channels using specific blockers may effectively suppress microglial proliferation and mitigated the neurotoxicity induced by Aβ-treated microglial cells [15]. Meanwhile, CLIC1 was positively correlated with infiltration of immune cells and significantly associated with worse overall survival in glioma patients[29]. CLIC1 is highly expressed in the cancer stem cells (CSCs) of glioblastoma, and modulates the tumour microenvironment, promoting cancer cell proliferation and invasion. Whereas, inhibition of CLIC1 significantly induces G1 phase cell cycle arrest in glioblastoma CSCs, and the functional impairment of CLIC1 on the membrane causes glioblastoma CSCs to shift from an allostatic tumorigenic state to a homeostatic steady state [30]. All these findings suggest that CLIC1 is a critical therapeutic target for central nervous system disorders and holds promising potential for treating CIRI. Furthermore, therapeutic regimens should aim to control CLIC1 temporally or spatially, adapting treatment to the disease stage or severity. The goal of targeted therapy is to inhibit its deleterious effects without completely abolishing its physiological function.

Comment #5

The experiment in Fig. 4 using activated NLRP3 after silencing CLIC1 to determine if NLRP3 mediates pyroptosis in OGD/R seems forced and superfluous since in the same Figure it was shown that NLRP3 is activated by OGD/R itself followed by pyroptosis markers. It is also merely strange to state in the same Fig legend (line 4-5) that activation of NLRP3 upregulates itself!

We thank the reviewer for their careful analysis of the design and interpretation of the experiments in Figure 4.

1. **The necessity of** **NLRP3 activation after CLIC1 silencing:**

**Our aim with this experiment was to clarify the mechanistic sequence and establish that CLIC1 acts upstream of NLRP3 in this pathway. By re-activating NLRP3 in CLIC1-silenced cells, we intended to determine whether pyroptosis could be restored, thus directly linking NLRP3 as the primary downstream effector of CLIC1 in OGD/R-induced injury.** We attempt to demonstrate that NLRP3 activation is sufficient to bypass CLIC1’s role, reinforcing NLRP3 as the central mediator. However, this could indeed appear redundant if OGD/R already activates NLRP3. **A better approach might have been to silence NLRP3 itself (not just CLIC1) to confirm its necessity. This will be refined in our future research.** We will rephrase and clarify in the manuscript in order to emphasis that the activation of NLRP3 is intended to reinforce the causal relationship between CLIC1 and NLRP3-mediated pyroptosis.

**Revised Discussion (end of paragraph 4)**

… We aimed to clarify the mechanistic relationship between CLIC1 and NLRP3 in OGD/R-induced injury by showing that re-activation of NLRP3 in CLIC1-silenced cells restores pyroptosis, supporting NLRP3 as the key downstream effector of CLIC1. However, since OGD/R alone activates NLRP3, silencing NLRP3 directly may have been a more definitive approach, and we will refine this aspect in future studies.

2. **Regarding the statement that "activation of NLRP3 upregulates itself":**
   We appreciate this being brought to our attention and agree that the current wording is misleading and scientifically inaccurate. Our intended meaning was that exogenous activation of NLRP3 in the context of CLIC1 silencing restores downstream pyroptosis markers, not that NLRP3 directly upregulates its own expression. In the revised legend of figure 4, we have corrected this statement for clarity.

 **Original Legend**

**Figure 4. CLIC1 promoted NLRP3-mediated pyroptosis via inhibiting Nrf2/HO-1 signalling pathway in OGD/R-treated HT22 cells.** a-c real-time PCR (a) and WB (b,c) analyses indicated that NLRP3 and caspase-1 expression were upregulated in OGD/R-treated HT22 cells and downregulated with CLIC1 silencing. They were also upregulated with inhibition of Nrf2/HO-1 or activation of NLRP3. d,e Expression levels of caspase-3 and GSDMD were upregulated in OGD/R-treated HT22 cells and downregulated with silencing of CLIC1 by both real-time PCR (d) and WB (e) analyses. f-h IL-1β, and IL-18 expression levels were upregulated in OGD/R-treated HT22 cells and downregulated with silencing of CLIC1 by both real-time PCR (f), WB (g) and ELISA(h) analyses. And all these inflammasomes were further upregulated with inhibition of Nrf2/HO-1 or activation of NLRP3 after CLICI silencing. i Protein expression of caspase-3, GSDMD, IL-1β, and IL-18 by WB. *n* =3 per group. ****P*<0.001, ***P*<0.01 vs control group*; ^###^P*<0.001, *^##^P*<0.01, *^#^P*<0.05 vs OGD/R group; ^&&&^*P*<0.001, ^&&^*P*<0.01, ^&^*P*<0.05 vs OGD/R+CLIC1 silencing group.

**Revised Figure 4 and legend**

**Fig 4. CLIC1 promotes NLRP3-mediated pyroptosis by inhibiting the Nrf2/HO-1 signalling pathway in oxygen and glucose deprivation/reoxygenation (OGD/R)-treated HT22 cells.** Real-time PCR, WB, and/or ELISA analyses show that the expression levels of inflammation- and pyroptosis-related indicators were all increased after inhibiting the Nrf2/HO-1 pathway OGD/R-treated HT22 cells. (a-c) Real-time PCR (a) and WB (b, c) analyses showed upregulation of NLRP3 and caspase-1 expression in OGD/R-treated HT22 cells, with significant downregulation upon CLIC1 silencing. Inhibition of Nrf2/HO-1 signalling or activation of NLRP3 partly reversed the effects of CLIC1 silencing. (d-f) WB analysis of caspase-3 and GSDMD protein expression showed similar trends with NLRP3 and caspase-1. (g-j) Real-time PCR (g), WB (h,i), and ELISA (j) analyses revealed elevated levels of pyroptosis-related inflammatory cytokines of IL-1β and IL-18 in OGD/R-treated HT22 cells, which were reduced upon CLIC1 silencing. These cytokines were further upregulated with Nrf2/HO-1 inhibition or NLRP3 activation after CLIC1 silencing. Quantitative data were presented as mean ± SEM with three replicate experiments. ^*^*P* <0.05 versus control group; **^^^***P* <0.05 versus OGD/R group; ^+^*P* <0.05 vs OGD/R+CLIC1 silencing group.

Comment #6

Page 4, 2nd pgph, line 5 : „CLIC1 ...removing excessive negative charge via anion channel“ However CLIC1 IS an anion channel

We have revised the corresponding statements in the article to enhance the rigour and readability of the article.

Chloride intracellular channel 1 (CLIC1) distributes in various organs and participates in enzymatic activity, redox regulation and inflammatory processes. It is identified as an oxidative-stress sensor in cells of the vascular endothelium[7]. CLIC1 was shown to be abundantly expressed in macrophages and attached to the cell membrane through oxidation and activation, removing the excessive negative charge via anion channels.

**Revised Introduction paragraph 3**

Chloride intracellular channel 1 (CLIC1) is a multifunctional protein expressed in various organs, where it participates in enzymatic activity, redox regulation and inflammatory processes [5, 6] . It is an oxidative stress sensor in vascular endothelium cells [7]. In response to increased cytoplasmic oxidation or changes in pH, cytoplasmic soluble CLIC proteins integrate into cellular membranes, functioning as anion channels to counteract cationic fluctuations [8]. CLIC1 was found to modulate cyclic adenosine monophosphate (cAMP)-induced chloride currents in bronchial epithelial cells, maintaining chloride homeostasis [9].

Comment #7

Legend to Fig. 1 is missing

Thank you for pointing out this shortage. We have provided additional clarification.

**Revised Figure 1 and supplemented legend**

**Fig 1. Schematic illustration of the hypothesis of CLIC1 mediates neuronal injury following OGD/R in HT22 cells.**

Mouse hippocampal neurons (modelled using HT22 cells) are subjected to OGD/R to mimic CIRI. OGD/R triggers upregulation of CLIC1, which leads to suppression of the Nrf2/HO-1 antioxidant signaling pathway. This results in increased ROS accumulation and disturbance of redox balance, promoting apoptosis by increased Bax and decreased Bcl-2 expression. Additionally, elevated CLIC1 activates the NLRP3 inflammasome and caspase-1, inducing pyroptosis via upregulation of pyroptosis markers, such as caspase-3, GSDMD, IL-1β, and IL-18. Collectively, these processes contribute to neuronal cell damage after CIRI. OGD/R, oxygen-glucose deprivation/reoxygenation; CIRI, cerebral ischemia-reperfusion injury; CLIC1, chloride intracellular channel 1; Nrf2, nuclear factor erythroid 2–related factor 2; ARE, antioxidant response element; HO-1, heme oxygenase 1; ROS, reactive oxygen species; NLRP3, NOD-like receptor family pyrin domain containing 3; GSDMD, gasdermin D.

Comment #8

First pgph on page 14 should belong to the part of text with Figure 4 .

We thank the reviewer for their careful analysis of the interpretation of the experiments in Figure 4. We have modified the legend of Figure 4 as suggested.

**Original Legend**

**Figure 4. CLIC1 promoted NLRP3-mediated pyroptosis via inhibiting Nrf2/HO-1 signalling pathway in OGD/R-treated HT22 cells.** a-c real-time PCR (a) and WB (b,c) analyses indicated that NLRP3 and caspase-1 expression were upregulated in OGD/R-treated HT22 cells and downregulated with CLIC1 silencing. They were also upregulated with inhibition of Nrf2/HO-1 or activation of NLRP3. d,e Expression levels of caspase-3 and GSDMD were upregulated in OGD/R-treated HT22 cells and downregulated with silencing of CLIC1 by both real-time PCR (d) and WB (e) analyses. f-h IL-1β, and IL-18 expression levels were upregulated in OGD/R-treated HT22 cells and downregulated with silencing of CLIC1 by both real-time PCR (f), WB (g) and ELISA(h) analyses. And all these inflammasomes were further upregulated with inhibition of Nrf2/HO-1 or activation of NLRP3 after CLICI silencing. i Protein expression of caspase-3, GSDMD, IL-1β, and IL-18 by WB. *n* =3 per group. *****P*<0.001, ***P*<0.01 vs control group*; ^###^P*<0.001, *^##^P*<0.01, *^#^P*<0.05 vs OGD/R group; ^&&&^*P*<0.001, ^&&^*P*<0.01, ^&^*P*<0.05 vs OGD/R+CLIC1 silencing group.

**Original First pgph on page 14**

**CLIC1 inhibits the Nrf2/HO-1 signalling pathway to accelerate pyroptosis**

As explained above, the increased CLIC1 led to decreased Nrf2/HO-1 expression. We also found that by real-time PCR, WB, and/or ELISA analyses, the expression levels of inflammation- and pyroptosis-related indicators NLRP3, caspase-1, caspase-3, GSDMD, IL-1β, and IL-18 were all increased after inhibiting the Nrf2/HO-1 pathway in HT22 cells treated with OGD/R, and their levels were higher in the OGD/R+CLIC1 silencing+ Nrf2/HO-1 inhibition group than in the OGD/R+CLIC1 silencing group (Fig 4). Thus, NLRP3-mediated pyroptosis could be accelerated by inhibiting Nrf2/HO-1 signal pathway.

**Revised Figure 4 and legend**

**Fig 4. CLIC1 promotes NLRP3-mediated pyroptosis by inhibiting the Nrf2/HO-1 signalling pathway in oxygen and glucose deprivation/reoxygenation (OGD/R)-treated HT22 cells.** Real-time PCR, WB, and/or ELISA analyses show that the expression levels of inflammation- and pyroptosis-related indicators were all increased after inhibiting the Nrf2/HO-1 pathway OGD/R-treated HT22 cells. (a-c) Real-time PCR (a) and WB (b, c) analyses showed upregulation of NLRP3 and caspase-1 expression in OGD/R-treated HT22 cells, with significant downregulation upon CLIC1 silencing. Inhibition of Nrf2/HO-1 signalling or activation of NLRP3 partly reversed the effects of CLIC1 silencing. (d-f) WB analysis of caspase-3 and GSDMD protein expression showed similar trends with NLRP3 and caspase-1. (g-j) Real-time PCR (g), WB (h,i), and ELISA (j) analyses revealed elevated levels of pyroptosis-related inflammatory cytokines of IL-1β and IL-18 in OGD/R-treated HT22 cells, which were reduced upon CLIC1 silencing. These cytokines were further upregulated with Nrf2/HO-1 inhibition or NLRP3 activation after CLIC1 silencing. Quantitative data were presented as mean ± SEM with three replicate experiments. ^*^*P* <0.05 versus control group; **^^^***P* <0.05 versus OGD/R group; ^+^*P* <0.05 vs OGD/R+CLIC1 silencing group.

Comment #9

There are a lot of typos and English is below acceptance level (eg “A recent study discovered that in umbilical vein endothelial cells, CLIC1 high-expressed and impaired the ability of vascular cells to resist oxidative stress to speed up cellular senescence, whose inhibition conversely perform a protective effect on preventing cellular aging and disorder”)

As non-native English speakers, we acknowledge that the manuscript contained some language and grammatical misapplications, and we sincerely apologize for these issues. To address and resolve these problems, we have thoroughly reviewed and corrected the full text and **engaged a professional agency (Charlesworth, UK)** to refine the article further. This process has corrected linguistic and grammatical errors and significantly enhanced the overall readability of the manuscript.

eg. Orignal: A recent study discovered that in umbilical vein endothelial cells, CLIC1 high-expressed and impaired the ability of vascular cells to resist oxidative stress to speed up cellular senescence, whose inhibition conversely perform a protective effect on preventing cellular aging and disorder.

**Revised:** A recent study discovered that in umbilical vein endothelial cells, upregulation of CLIC1 impaired the ability of vascular cells to resist oxidative stress to speed up cellular senescence; in contrast, CLIC1 inhibition resulted in a protective effect, preventing cellular aging and disorder.

Comment #10

The authors claim that all relevant data are within the ms but this is not the case – jus the means and errors can be found.

Thank you for your careful reviewing, we have revised this as: **“All relevant data are within the manuscript and its Supporting Information files.”**

Comment #11

Why do the authors disclose ethical review of animal welfare when they used no animals in their experiments? Only commercially available animal cell line was used

Our experiments involve both animal and cellular experiments. In this paper, we have written only the part concerning cellular experiments. **We have removed the ethical statement about animal experiments in the revised manuscript.**

**References**

1.	Dejas L, Santoni K, Meunier E, Lamkanfi M. Regulated cell death in neutrophils: From apoptosis to NETosis and pyroptosis. Semin Immunol. 2023;70:101849.doi:10.1016/j.smim.2023.101849

2.	Li L, Dickinson M S, Coers J, Miao E A. Pyroptosis in defense against intracellular bacteria. Semin Immunol. 2023;69:101805.doi:10.1016/j.smim.2023.101805

3.	Yuan R, Xu H, Wang M, Guo L, Yao Y, Zhang X, et al. Promoting the transition from pyroptosis to apoptosis in endothelial cells: a novel approach to alleviate methylglyoxal-induced vascular damage. J Transl Med. 2025;23(1):170.doi:10.1186/s12967-025-06195-x

4.	Cao Y, Hu L, Chen R, Chen Y, Liu H, Wei J. Unfolded protein response-activated NLRP3 inflammasome contributes to pyroptotic and apoptotic podocyte injury in diabetic kidney disease via the CHOP-TXNIP axis. Cell Signal. 2025;130:111702.doi:10.1016/j.cellsig.2025.111702

5.	Salao K, Jiang L, Li H, Tsai V W, Husaini Y, Curmi P M, et al. CLIC1 regulates dendritic cell antigen processing and presentation by modulating phagosome acidification and proteolysis. Biology Open. 2016;5(5):620-630.doi:10.1242/bio.018119

6.	Al Khamici H, Brown L J, Hossain K R, Hudson A L, Sinclair-Burton A A, Ng J P, et al. Members of the chloride intracellular ion channel protein family demonstrate glutaredoxin-like enzymatic activity. PLoS One. 2015;10(1):e115699.doi:10.1371/journal.pone.0115699

7.	Lu D, Le Y, Ding J, Dou X, Mao W, Zhu J. CLIC1 Inhibition Protects Against Cellular Senescence and Endothelial Dysfunction Via the Nrf2/HO-1 Pathway. Cell Biochem Biophys. 2021;79(2):239-252.doi:10.1007/s12013-020-00959-6

8.	Domingo-Fernandez R, Coll R C, Kearney J, Breit S, O'Neill L A J. The intracellular chloride channel proteins CLIC1 and CLIC4 induce IL-1beta transcription and activate the NLRP3 inflammasome. Journal of Biological Chemistry. 2017;292(29):12077-12087.doi:10.1074/jbc.M117.797126

9.	Liu B, Billington C K, Henry A P, Bhaker S K, Kheirallah A K, Swan C, et al. Chloride intracellular channel 1 (CLIC1) contributes to modulation of cyclic AMP-activated whole-cell chloride currents in human bronchial epithelial cells. Physiological Reports. 2018;6(2).doi:10.14814/phy2.13508

10.	Alzaydi M M, Abdul-Salam V B, Whitwell H J, Russomanno G, Glynos A, Capece D, et al. Intracellular Chloride Channels Regulate Endothelial Metabolic Reprogramming in Pulmonary Arterial Hypertension. American Journal of Respiratory Cell and Molecular Biology. 2023;68(1):103-115.doi:10.1165/rcmb.2022-0111OC

11.	Sun X, Cui X. Isorhapontigenin alleviates cerebral ischemia/reperfusion injuries in rats and modulated the PI3K/Akt signaling pathway. Naunyn-Schmiedeberg's Archives of Pharmacology. 2020;393(9):1753-1760.doi:10.1007/s00210-019-01794-0

12.	Zhou S, Gao X, Chen C, Zhang J, Zhang Y, Zhang L, et al. Porcine cardiac blood - Salvia miltiorrhiza root alleviates cerebral ischemia reperfusion injury by inhibiting oxidative stress induced apoptosis through PI3K/AKT/Bcl-2/Bax signaling pathway. Journal of Ethnopharmacology. 2023;316:116698.doi:10.1016/j.jep.2023.116698

13.	Carlini V, Verduci I, Cianci F, Cannavale G, Fenoglio C, Galimberti D, et al. CLIC1 Protein Accumulates in Circulating Monocyte Membrane during Neurodegeneration. International Journal of Molecular Sciences. 2020;21(4).doi:10.3390/ijms21041484

14.	Barbieri F, Verduci I, Carlini V, Zona G, Pagano A, Mazzanti M, et al. Repurposed Biguanide Drugs in Glioblastoma Exert Antiproliferative Effects via the Inhibition of Intracellular Chloride Channel 1 Activity. Frontiers in Oncology. 2019;9:135.doi:10.3389/fonc.2019.00135

15.	Luo L, Song S, Ezenwukwa C C, Jalali S, Sun B, Sun D. Ion channels and transporters in microglial function in physiology and brain diseases. Neurochemistry International. 2021;142:104925.doi:10.1016/j.neuint.2020.104925

16.	Djuric U, Lam K H B, Kao J, Batruch I, Jevtic S, Papaioannou M D, et al. Defining Protein Pattern Differences Among Molecular Subtypes of Diffuse Gliomas Using Mass Spectrometry. Molecular & Cellular Proteomics. 2019;18(10):2029-2043.doi:10.1074/mcp.RA119.001521

17.	Francisco M A, Wanggou S, Fan J J, Dong W, Chen X, Momin A, et al. Chloride intracellular channel 1 cooperates with potassium channel EAG2 to promote medulloblastoma growth. Journal of Experimental Medicine. 2020;217(5).doi:10.1084/jem.20190971

18.	Gururaja Rao S, Patel N J, Singh H. Intracellular Chloride Channels: Novel Biomarkers in Diseases. Frontiers In Physiology. 2020;11:96.doi:10.3389/fphys.2020.00096

19.	Lee J R, Lee J Y, Kim H J, Hahn M J, Kang J S, Cho H. The inhibition of chloride intracellular channel 1 enhances Ca(2+) and reactive oxygen species signaling in A549 human lung cancer cells. Exp Mol Med. 2019;51(7):1-11.doi:10.1038/s12276-019-0279-2

20.	Ko M, Jung H Y, Lee D, Jeon J, Kim J, Baek S, et al. Inhibition of chloride intracellular channel protein 1 (CLIC1) ameliorates liver fibrosis phenotype by activating the Ca(2+)-dependent Nrf2 pathway. Biomedicine & Pharmacotherapy. 2023;168:115776.doi:10.1016/j.biopha.2023.115776

21.	Luo J F, Shen X Y, Lio C K, Dai Y, Cheng C S, Liu J X, et al. Activation of Nrf2/HO-1 Pathway by Nardochinoid C Inhibits Inflammation and Oxidative Stress in Lipopolysaccharide-Stimulated Macrophages. Frontiers in Pharmacology. 2018;9:911.doi:10.3389/fphar.2018.00911

22.	Chen G H, Song C C, Pantopoulos K, Wei X L, Zheng H, Luo Z. Mitochondrial oxidative stress mediated Fe-induced ferroptosis via the NRF2-ARE pathway. Free Radic Biol Med. 2022;180:95-107.doi:10.1016/j.freeradbiomed.2022.01.012

23.	Zhang Q, Wang J, Zhang H, Zeng T. Dihydromyricetin inhibits oxidative stress and apoptosis in oxygen and glucose deprivation/reoxygenation‑induced HT22 cells by activating the Nrf2/HO‑1 pathway. Molecular Medicine Reports. 2021;23(6).doi:10.3892/mmr.2021.12036

24.	Wang L, Zhang X, Xiong X, Zhu H, Chen R, Zhang S, et al. Nrf2 Regulates Oxidative Stress and Its Role in Cerebral Ischemic Stroke. Antioxidants (Basel). 2022;11(12).doi:10.3390/antiox11122377

25.	Swanson K V, Deng M, Ting J P. The NLRP3 inflammasome: molecular activation and regulation to therapeutics. Nature Reviews Immunology. 2019;19(8):477-489.doi:10.1038/s41577-019-0165-0

26.	Xu M S, Yin L M, Cheng A F, Zhang Y J, Zhang D, Tao M M, et al. Cerebral Ischemia-Reperfusion Is Associated With Upregulation of Cofilin-1 in the Motor Cortex. Frontiers in Cell and Developmental Biology. 2021;9:634347.doi:10.3389/fcell.2021.634347

27.	Zhang M, Liu Z, Zhou W, Shen M, Mao N, Xu H, et al. Ferrostatin-1 attenuates hypoxic-ischemic brain damage in neonatal rats by inhibiting ferroptosis. Translational Pediatrics. 2023;12(11):1944-1970.doi:10.21037/tp-23-189

28.	Chae B J, Lee K S, Hwang I, Yu J W. Extracellular Acidification Augments NLRP3-Mediated Inflammasome Signaling in Macrophages. immune-network. 2023;23(3):e23.doi:10.4110/in.2023.23.e23

29.	Liu C, Zhang W, Xu G, Zhang D, Zhang C, Qiao S, et al. Deep multilayer brain omics identifies the potential involvement of menopause molecular networks in Gliomas' disease progression. FASEB Journal. 2022;36(10):e22570.doi:10.1096/fj.202200427RR

30.	Peretti M, Raciti F M, Carlini V, Verduci I, Sertic S, Barozzi S, et al. Mutual Influence of ROS, pH, and CLIC1 Membrane Protein in the Regulation of G(1)-S Phase Progression in Human Glioblastoma Stem Cells. Molecular Cancer Therapeutics. 2018;17(11):2451-2461.doi:10.1158/1535-7163.MCT-17-1223

31.	Li M, Tang H, Li Z, Tang W. Emerging Treatment Strategies for Cerebral Ischemia-Reperfusion Injury. Neuroscience. 2022;507:112-124.doi:10.1016/j.neuroscience.2022.10.020

32.	Zhang M, Liu Q, Meng H, Duan H, Liu X, Wu J, et al. Ischemia-reperfusion injury: molecular mechanisms and therapeutic targets. Signal Transduction and Targeted Therapy. 2024;9(1):12.doi:10.1038/s41392-023-01688-x

33.	Pell V R, Spiroski A M, Mulvey J, Burger N, Costa A S H, Logan A, et al. Ischemic preconditioning protects against cardiac ischemia reperfusion injury without affecting succinate accumulation or oxidation. J Mol Cell Cardiol. 2018;123:88-91.doi:10.1016/j.yjmcc.2018.08.010

34.	Xu Q, Cheung R T F. Melatonin at repeated doses alleviates hyperglycemia-exacerbated cerebral ischemia-reperfusion injury at 72 h via anti-inflammation and anti-apoptosis. Ibro Neuroscience Reports. 2024;16:418-427.doi:10.1016/j.ibneur.2024.03.003
