# Supplementary material for: CLIC1 down-regulates Nrf2/HO-1 signalling pathway promoting the apoptosis and pyroptosis in OGD/R-treated HT22 cells
Source: PLoS One. 2025 Sep 18;20(9):e0332698. doi: 10.1371/journal.pone.0332698 (PMC12445528; doi:10.1371/journal.pone.0332698)
Supplement: Attachment — Submitted filename: Response to Reviewers_2025.9.1.docx [file pone.0332698.s011.docx]

**Dear Editor and Reviewer,**

We would like to express our sincere gratitude for your time, effort, and constructive feedback throughout the review process. Your insightful comments and valuable suggestions have been instrumental in helping us improve the quality and clarity of our manuscript.

To facilitate your review, we have:

- Provided a detailed, point-by-point response to all comments in this letter.
- Highlighted all relevant modifications to the main text in yellow.

We deeply appreciate your careful evaluation and support, which have contributed significantly to strengthening our work. Thank you again for your guidance and for the opportunity to revise and resubmit our article.

Please do not hesitate to contact us if you have any further questions or concerns.

Best regards,

Chuang Sun

**Journal requirements:**

Comment #1

If the reviewer comments include a recommendation to cite specific previously published works, please review and evaluate these publications to determine whether they are relevant and should be cited. There is no requirement to cite these works unless the editor has indicated otherwise. Please review your reference list to ensure that it is complete and correct. If you have cited papers that have been retracted, please include the rationale for doing so in the manuscript text, or remove these references and replace them with relevant current references. Any changes to the reference list should be mentioned in the rebuttal letter that accompanies your revised manuscript. If you need to cite a retracted article, indicate the article’s retracted status in the References list and also include a citation and full reference for the retraction notice.

Thank you for your note regarding the citations. In response to the reviewers’ and editor’s comments, we have carried out the following verifications and amendments:

1. We have individually cross-checked all references to ensure the accuracy of cited content, and verified whether any articles have been retracted or removed from the Science Citation Index (up to 15 August 2025).

2. One additional article that had been removed from the SCI has been deleted from our reference list:

11.	Shi S, Zhang C, Liu J. TIMP2 facilitates CIRI through activating NLRP3-mediated pyroptosis. Aging (Albany New York). 2023;15(9):3635-3643.doi:10.18632/aging.204696

3. Reference 48 (Zhao M W, Yang P, Zhao L L. *Environmental Toxicology*. 2019) has been deleted due to limited relevance to the current manuscript.

48.	Zhao M W, Yang P, Zhao L L. Chlorpyrifos activates cell pyroptosis and increases susceptibility on oxidative stress-induced toxicity by miR-181/SIRT1/PGC-1alpha/Nrf2 signaling pathway in human neuroblastoma SH-SY5Y cells: Implication for association between chlorpyrifos and Parkinson's disease. Environmental Toxicology. 2019;34(6):699-707.doi:10.1002/tox.22736

4. In accordance with Reviewer 3’s new comments, we have removed certain tumour-related references and supplemented the manuscript with additional literature relevant to stroke and Alzheimer’s disease. Further details are included in our point-by-point responses to the reviewer comments below.

Comment #2

While revising your submission, please upload your figure files to the Preflight Analysis and Conversion Engine (PACE) digital diagnostic tool, <https://pacev2.apexcovantage.com/>. PACE helps ensure that figures meet PLOS requirements.

We have uploaded all figure files to the Preflight Analysis and Conversion Engine (PACE) digital diagnostic tool, as recommended. This ensures that all images comply with the journal's publication requirements.

**Response to reviewer #3:**

Comments are addressed thoroughly and successfully.

Thank you for your positive feedback. We appreciate your acknowledgement and are glad that our revisions have addressed your privous comments satisfactorily.

Moreover, we are grateful for your additional suggestions. We will address and amend them point by point as outlined below. For your convenience, all relevant amendments within the main text have been highlighted in yellow. Your valuable insights have further enhanced the quality and rigour of our article.

Comment #1

Nevertheless, it would be more appropriate in the revised ms to use examples from CNS and not from cancer (Discussion Pgph 6) or vascular and bronchial tissues (Introduction pgph 3).

**1. Introduction**

Paragraph 3 original:

CLIC1 was found to modulate cyclic adenosine monophosphate (cAMP)-induced chloride currents in bronchial epithelial cells, maintaining chloride homeostasis [1].

Thank you for your observation. We have removed the reference to CLIC1’s role in bronchial epithelial cells from Introduction Paragraph 3, as it was not directly relevant to CNS disorders.

**2. Discussion**

Thank you for your valuable feedback. We have taken your suggestions into consideration and, given the specialized nature of oncology, have removed the literature pertaining to brain tumors. Instead, we have supplemented our review with research on other central nervous system disorders, including stroke and Alzheimer’s disease.

Paragraph 2 original:

CLIC1 is a metamorphic protein that exists in both soluble cytoplasmic and membrane-associated forms, with the latter functioning as a chloride-selective ion channel[2]…. Previous research has reported elevated CLIC1 levels in various neurological disorders and central nervous system cancers, such as Alzheimer’s disease (AD), medulloblastoma, and gliomas [3-5]….

Revised:

CLIC1 is a metamorphic protein that exists in both soluble cytoplasmic and membrane-associated forms, with the latter functioning as a chloride-selective ion channel[2]…. Previous research has reported elevated CLIC1 levels in various neurological disorders and central nervous system cancers, such as Alzheimer’s disease and stroke [5, 6]. For example, CLIC1 upregulates in CD8 T cells and natural killer cells in atrial fibrillation thrombi, indicating its potential involvement in cytotoxic activity and tissue injury, thereby posing a risk of cardioembolic stroke[6]….

Paragraph 6 original:

Currently, … Although there are no specific studies directly focusing on CLIC1 inhibitors for CIRI, previous studies [7, 8] have suggested CLIC1 as a potential therapeutic target for central nervous system diseases, such as brain tumours, neurodegenerative diseases, and ischemic stroke. For example, inhibition of CLIC1 channels may effectively suppress microglial proliferation and mitigated the neurotoxicity induced by Aβ-treated microglial cells [7]. Meanwhile, CLIC1 was positively correlated with infiltration of immune cells and significantly associated with worse overall survival in glioma patients[8]. CLIC1 is highly expressed in the cancer stem cells (CSCs) of glioblastoma, and modulates the tumour microenvironment, promoting cancer cell proliferation and invasion. Whereas, inhibition of CLIC1 significantly induces G1 phase cell cycle arrest in glioblastoma CSCs, and the functional impairment of CLIC1 on the membrane causes glioblastoma CSCs to shift from an allostatic tumorigenic state to a homeostatic steady state [9]. All these findings suggest that CLIC1 is a critical therapeutic target for central nervous system disorders and holds promising potential for treating CIRI. …

Revised:

Currently, … Although there are no specific studies directly focusing on CLIC1 inhibitors for CIRI, previous studies [6, 7, 10] have suggested CLIC1 as a potential therapeutic target for central nervous system diseases, such as neurodegenerative diseases and ischemic stroke. For example, inhibition of CLIC1 channels may effectively suppress microglial proliferation and mitigated the neurotoxicity induced by Aβ-treated microglial cells [7]. Furthermore, a recent study[10] elucidates the regulatory role of circAPP in Alzheimer's disease via the miR-1906/CLIC1 axis during microglial polarisation. All these findings suggest that CLIC1 is a critical therapeutic target for central nervous system disorders and holds promising potential for treating CIRI….

Comment #2

In addition, text in Duscussion pgph 2 5th line from the end should be changed to read "...highlighting its role in the apoptotic process..."

Thank you for identifying the grammatical errors in our article. We have made the necessary corrections in the relevant sections.

**Discussion**

Paragraph 2 original:

Furthermore, we observed that CLIC1 regulated apoptotic markers, such as the pro-apoptotic Bax and anti-apoptotic Bcl-2, highlight its role in apoptosis process in OGD/R-treated HT22 cells.

Revised:

Furthermore, we observed that CLIC1 regulated apoptotic markers, such as the pro-apoptotic Bax and anti-apoptotic Bcl-2, highlighting its role in apoptosis process in OGD/R-treated HT22 cells.

**References**

1.	Liu B, Billington C K, Henry A P, Bhaker S K, Kheirallah A K, Swan C, et al. Chloride intracellular channel 1 (CLIC1) contributes to modulation of cyclic AMP-activated whole-cell chloride currents in human bronchial epithelial cells. Physiological Reports. 2018;6(2).doi:10.14814/phy2.13508

2.	Barbieri F, Verduci I, Carlini V, Zona G, Pagano A, Mazzanti M, et al. Repurposed Biguanide Drugs in Glioblastoma Exert Antiproliferative Effects via the Inhibition of Intracellular Chloride Channel 1 Activity. Frontiers in Oncology. 2019;9:135.doi:10.3389/fonc.2019.00135

3.	Djuric U, Lam K H B, Kao J, Batruch I, Jevtic S, Papaioannou M D, et al. Defining Protein Pattern Differences Among Molecular Subtypes of Diffuse Gliomas Using Mass Spectrometry. Molecular & Cellular Proteomics. 2019;18(10):2029-2043.doi:10.1074/mcp.RA119.001521

4.	Francisco M A, Wanggou S, Fan J J, Dong W, Chen X, Momin A, et al. Chloride intracellular channel 1 cooperates with potassium channel EAG2 to promote medulloblastoma growth. Journal of Experimental Medicine. 2020;217(5).doi:10.1084/jem.20190971

5.	Gururaja Rao S, Patel N J, Singh H. Intracellular Chloride Channels: Novel Biomarkers in Diseases. Frontiers In Physiology. 2020;11:96.doi:10.3389/fphys.2020.00096

6.	Renedo D, Barak T, DeLong J, Acosta J N, Sujijantarat N, Koo A, et al. Characterizing Stroke Clots Using Single-Cell Sequencing. Journal of the American Heart Association. 2025:eJAHA2025041738T.doi:10.1161/JAHA.125.041738

7.	Luo L, Song S, Ezenwukwa C C, Jalali S, Sun B, Sun D. Ion channels and transporters in microglial function in physiology and brain diseases. Neurochemistry International. 2021;142:104925.doi:10.1016/j.neuint.2020.104925

8.	Liu C, Zhang W, Xu G, Zhang D, Zhang C, Qiao S, et al. Deep multilayer brain omics identifies the potential involvement of menopause molecular networks in Gliomas' disease progression. FASEB Journal. 2022;36(10):e22570.doi:10.1096/fj.202200427RR

9.	Peretti M, Raciti F M, Carlini V, Verduci I, Sertic S, Barozzi S, et al. Mutual Influence of ROS, pH, and CLIC1 Membrane Protein in the Regulation of G(1)-S Phase Progression in Human Glioblastoma Stem Cells. Molecular Cancer Therapeutics. 2018;17(11):2451-2461.doi:10.1158/1535-7163.MCT-17-1223

10.	Wu D P, Wei Y S, Hou L X, Du Y X, Yan Q Q, Liu L L, et al. Circular RNA APP contributes to Alzheimer's disease pathogenesis by modulating microglial polarization via miR-1906/CLIC1 axis. Alzheimers Res Ther. 2025;17(1):44.doi:10.1186/s13195-025-01698-7
